# Predicting genome terminus sequences of *Bacillus cereus*-group bacteriophage using next generation sequencing data

**DOI:** 10.1186/s12864-017-3744-0

**Published:** 2017-05-04

**Authors:** Cheng-Han Chung, Michael H. Walter, Luobin Yang, Shu-Chuan (Grace) Chen, Vern Winston, Michael A. Thomas

**Affiliations:** 1 Department of Biological Sciences, Idaho State University, 921 South 8th Avenue, Pocatello, ID 83209-8007 USA; 2 Department of Biology, University of Northern Iowa, 144 McCollum Science Hall, Cedar Falls, IA 50614-0421 USA; 3 Department of Mathematics and Statistics, Idaho State University, 921 South 8th Avenue, Pocatello, ID 83209-8085 USA

**Keywords:** Bacteriophage, Phage genome configuration, Direct terminal repeat, Genome packaging mechanisms, Terminus prediction, Neighboring coverage ratio, Read edge frequency

## Abstract

**Background:**

Most tailed bacteriophages (phages) feature linear dsDNA genomes. Characterizing novel phages requires an understanding of complete genome sequences, including the definition of genome physical ends.

**Result:**

We sequenced 48 *Bacillus cereus* phage isolates and analyzed Next-generation sequencing (NGS) data to resolve the genome configuration of these novel phages. Most assembled contigs featured reads that mapped to both contig ends and formed circularized contigs. Independent assemblies of 31 nearly identical I48-like *Bacillus* phage isolates allowed us to observe that the assembly programs tended to produce random cleavage on circularized contigs. However, currently available assemblers were not capable of reporting the underlying phage genome configuration from sequence data. To identify the genome configuration of sequenced phage *in silico*, a terminus prediction method was developed by means of ‘neighboring coverage ratios’ and ‘read edge frequencies’ from read alignment files. Termini were confirmed by primer walking and supported by phylogenetic inference of large DNA terminase protein sequences.

**Conclusions:**

The Terminus package using phage NGS data along with the contig circularity could efficiently identify the proximal positions of phage genome terminus. Complete phage genome sequences allow a proposed characterization of the potential packaging mechanisms and more precise genome annotation.

**Electronic supplementary material:**

The online version of this article (doi:10.1186/s12864-017-3744-0) contains supplementary material, which is available to authorized users.

## Background

Tailed, double-stranded DNA bacteriophages (phages) are the most abundant type of phage described in the literature [1, 2]. Tailed phages share similar mechanisms for DNA packaging, but the diversity of genome configuration suggests that DNA recognition and cleavage mechanisms may differ [3, 4]. DNA packaging motor comprises of three components including the large terminase subunit (TerL), small terminase subunit (TerS) and Portal proteins, which largely dictates the packaging strategies (reviewed in [5, 6]). Packaging strategies are classified based on differing genomic termini. Phages lambda and P2 have 5′ cohesive ends [7, 8]. Mycobacteriophage L5 and D29 [9, 10], *Bacillus subtilis* phage phi105 [11] and *E. coli* phage HK97 have 3′ extensions [12]. Circularly permuted headful packaging systems are used by phages such as P22, SPP1 and T4 [13–15]. Phages T3 and T7 have non-permuted and relatively short direct terminal repeats with exactly the same length DNA in every virion genome [16, 17]. In contrast, various lengths of long terminal repeats are found in T5 and SPO1 [18, 19]. N4-like phages feature dynamic lengths of terminal repeats [20]. Phi29-like phages have covalently bound terminal proteins [21, 22]. Headful packaging is evident in Mu-like phages with host DNA sequence flanking the integrating position of host chromosome [23–25]. A collection of diverse types of genome termini has been described elsewhere [26, 27]. DNA motifs on phage genomes such as *cos* and *pac* site have been identified, which play crucial in the interaction with packaging motor and the subsequent product of genome configuration with distinguishable terminus feature mentioned above [16, 17, 28–31].

The high throughput volume of NGS allows high sequencing coverage of phage genomes and can reveal genome characteristics such as genome sequence redundancy and cleavage sites at genome ends. Yee and colleagues identified the build-up of coverage in the middle of coverage maps in the whole genome sequence of SPO1-similar phage SP10 [32]. They defined the region of higher read depth as genome terminal redundancy with approximately 12 kb [32]. However, they did not describe the characteristics of the terminal sequence. NGS read frequencies (number of reads with identical length and mapping positions on contig) revealed that T4-like phage IME08, which has a circularly permuted genome, has a sequence preference at the genome terminus rather than at a random cleavage site during headful packaging [33].

In 2012, Gill and colleagues characterized five *Caulobacter crescentus* phages that are closely related to phiCbK [34]. The NGS analysis of phiCbK-related phages showed 10–17 kb terminal redundancies based on a striking high coverage region over the assembled contig. The genomic terminal redundancies were further confirmed by tagging the genome termini with short nucleotide fragment as sequencing markers before high throughput sequencing [34]. Li and others also tagged genome termini of T3 phage with ligated adaptors for high throughput sequencing to locate the terminus. A set of lytic phages isolated from sewage were NGS-sequenced and characterized to determine termini based on nucleotides having the highest read frequencies [35]. However, the high frequency reads may vary in genome ends among phages and NGS sequencing methods. A recent study described a modification of Phamarator program to construct a customized database for comparative analysis of similar bacteriophages [36]. Merrill et al. used a Pile-up Analysis Using Starts & Ends (PAUSE) program (https://cpt.tamu.edu/computer-resources/pause) and a mapped reads visualization tool called Consed to identify the physical ends [37]. PAUSE identifies peaks of coverage build-up based on Continuous Wavelet Transform. PAUSE analyzes sense and antisense read density separately while our methods aggregate all mapped reads regardless of read orientations. Consed provides good visualization of regions of coverage decrease that imply physical ends of phage genomes. However, Consed does not implement quantitative methods to identify the physical ends. Recently, Zhang et al. developed the termini analysis theory to identify the protruding ends of newly sequenced E. faecium phages, which did not apply on other terminus forms but 3′ cohesive terminus [38].

In the present study, we calculated the 5′ or 3′ read edge frequency, and neighboring coverage ratios (NCR) to predict the positions of genome termini on a given contig. Raw sequencing data from nine published phages were used to calibrate the accuracy of the terminus-determining method in this study. Phylogenetic analysis of terminase large subunit genes and ‘primer walking’ using Sanger sequencing were also conducted to validate the putative termini from NGS data for the isolates sequenced. A study of a *Bacillus anthracis* phage assemblage characterized three novel phages from urban Iowa topsoil [39]. One spore-adhering phage named SBP8a has also been scrutinized in detail [40]. Here, we characterized genomic DNA of 48 naturally occurring phages that can infect *Bacillus anthracis/cereus* from the same soil source. We also re-interrogated NGS data of SBP8a.

The read edge frequencies and NCR are proposed as two important criteria to predict the genome termini for reconstructing complete physical maps for novel phages.

## Methods

### Culture media and bacteria strains

Host *Bacillus anthracis* Sterne and *Bacillus cereus* 569 UM20 were originally provided by Dr. J. Jackman’s lab (Johns Hopkins University Applied Physics Laboratory, Laurel, MD) and Dr. Terri Koehler’s lab (Department of Microbiology and Molecular Genetics, University of Texas-Houston Medical School, Houston, TX), respectively. The ‘safe strain’ *B. anthracis* Sterne is a vaccine strain without virulent plasmids pXO1 and pXO2. Tryptic Soy Broth (TSB, Difco Bacto BBL 211824) and solid media plates (1.5% Bacto-Agar) were used for growth in this study.

### Isolation, propagation/increase and DNA extraction of phages

Natural *B. anthracis* phages were isolated and purified as previously described [39]. Culture lysis, lysate clarification, triple-serial transfer and isolation were conducted by standard phage methods [41]. All *B. anthracis* phages were isolated and purified with the same procedure as SBP8a isolate [40]. Phage DNA was extracted by methods previously described [42]. A 100 ng aliquot of genomic DNA from each isolate was used to perform high throughput sequencing.

### Genomic DNA sequencing

Phage genomic DNA was sequenced by *Ion Torrent PGM* 316 chip v2 (*Life Technologies*, CA, USA) and by *MiSeq* Reagent Kit 2x300 v3 (*Illumina*, San Diego, CA, USA) at the Molecular Research Core Facility (Idaho State University, ID, USA).

For *PGM*, a DNA library was prepared by protocol of the *Ion Xpress* ^*TM*^ *Plus Fragment Library Kit* with Barcode Adapters 1–96 Kit (*Life Technologies*, CA, USA). For *MiSeq*, a library was prepared similarly, using the *Nextera XT DNA Sample Preparation Kit* and barcoded with the *MiSeq* Index Kit (*Illumina*, San Diego, CA, USA). Sequencing data of SBP8a phage by *Roche/454* technology was shared by Dr. Ian Molineux (Section of Molecular Genetics and Microbiology, University of Texas at Austin, TX, USA) and was also included as a replicate of SBP8a genome.

Nine published genome sequences and NGS reads of characterized phages were obtained from Dr. Graham Hatfull’s lab (Department of Biological Sciences, University of Pittsburg, PA, USA), including three *Bacillus* phages Adelynn (*MiSeq*), Nigalana [KU737344.1] (*Roche/454*) and Troll [KF208639.2] (*PGM*); three Cluster C mycobacteriophages Zeenon [KT321476] (*MiSeq*), Teardrop (*Roche/454*) and Breeniome [KF006817] (*PGM*) [43]; three Cluster A mycobacteriophages Equemioh13 [KJ959632] (*MiSeq*), Zetzy (*Roche/454*) and Lilith (*PGM*). The accession numbers are listed in brackets while available in GenBank.

### Criteria of predicted genome termini

NCR and read edge frequencies were used for investigating phage genomic termini, which are described in detail below. Predicting termini from mapped reads must surpass twin criteria: high read edge frequency and local highest/lowest NCR.

**Fig. 1:**
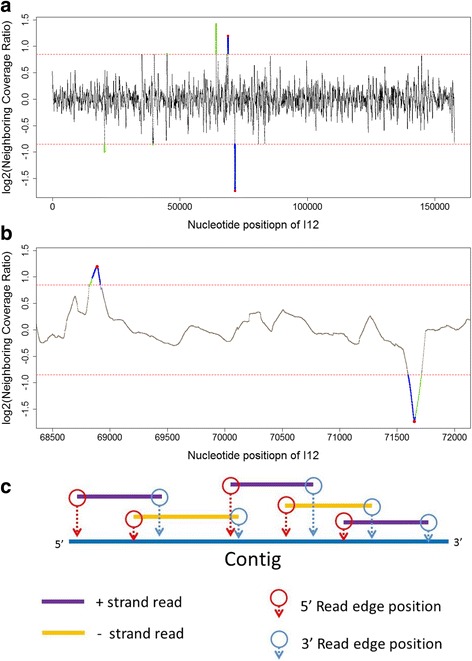
Illustration of two major characteristics of phage genome sequencing used for terminus prediction: Neighboring coverage ratio (NCR) and read edge frequency. I12 phage was used as an example of the selection process of the NCRs that are considered as potential boundaries of terminal repeats. Each *dot* represents the logarithmic transformed NCR on given nucleotide position with 100-nucleotide window size. Two *horizontal dashed lines* show the threshold of 1.8 NCR and reciprocal of 1.8. NCRs that are greater than 1.8 or less than reciprocal of 1.8 are collected in a subset of hits (*green dots*). Within the subset, hits with at least one window coverage of given NCR is 1.8 times greater than genome coverage are considered as significant hits (*blue dots*). Finally, the local highest and local lowest of significant hits are considered as potential boundaries of terminal repeats (*red dots*). **a** The whole-contig NCR of I12 isolate. **b** The NCR between nucleotide position 68,500 and 72,000. **c** Every mapped read has one corresponding coordinate at its 5′ end (5′ read edge position) and one at 3′ end (3′ read edge position). The counts of every read edge position were used as one of the indicators of terminus prediction

NCRs were calculated by the coverage within a 100-nucleotide section (sliding ‘window size’) to the right of any base coordinate (‘downstream window’) divided by coverage within an adjacent 100 nucleotide window to the left (‘upstream window’). The size of the window sliding step was one nucleotide. Each NCR was recorded on the first nucleotide of a downstream window. The NCRs that met the following criteria were considered as ‘significant hits’: (1) An NCR value greater than 1.8 followed by a NCR that is less than reciprocal of 1.8; (2) At least one window coverage (upstream or downstream) was 1.8 times greater than the average genome coverage, which indicated that the sliding windows were located within a high coverage region (Fig. 1a). Due to the assumption that the repeat region will be sequence twice as much as other genomic regions, fold-change of 1.8 was assigned empirically that allowed the read number variation sampled at genome termini. Among potential hits, the corresponding positions of local highest and local lowest NCR were considered as potential boundaries of terminal repeats (Fig. 1b).

Potential termini were also examined by read edge positions and corresponding frequencies. In the present study, the relative nucleotide positions of a given read were acquired based on contig position. The 5′ end and 3′ end of a read based on contig position were defined as the 5′ read edge position and the 3′ read edge position, respectively (Fig. 1c). The frequencies of read edge positions were calculated from all mappable reads. The top three frequencies of 5′ and 3′ edge positions were listed as potential termini of the genome.

A nucleotide position that was a potential boundary of terminal repeats in NCR screening and that had high read edge frequency was considered as a ‘predicted terminus’. More discussion and results are described in Prediction of genomic termini by NGS data in detail.

### Bioinformatics Analysis

Genomic contigs were assembled with Newbler 2.9 (*454 Life Sciences*) and Velvet 1.2.10 [44] for sequence reads from *PGM* and *MiSeq*, respectively. Among the contig sequences of I48-like isolates, over-call (insertion) and under-call (deletion) of homopolymers from *PGM* and/or *MiSeq* were corrected based on the majority call on the aligned position when homopolymers of more than two bases from *MiSeq* sequences varied in *PGM* sequences.

The NCRs, read edge frequencies, judgment of circular/linear contig and flanking sequence were analyzed with *perl* script Terminus.SE and Terminus.PE (https://github.com/james0032/Terminus). These scripts incorporated *Bowtie2* and the *samtools* package as part of the pipeline for terminus prediction [45, 46]. *Weblogo 3.4* was used to visualize read sequence conservation [47]. Open reading frames (ORFs) were predicted with both *Glimmer 3.02* and *GeneMarkS* [48, 49]. The search for homologous proteins and protein function of each predicted ORF was conducted by BLASTP (comparing against non-redundant database in NCBI) and *HHblits* (HMM-HMM-based lightning-fast iterative sequence search, HHsuite 2.0; http://toolkit.genzentrum.lmu.de/hhblits/) comparing against the uniprot20 database. Amino acid sequences of the large terminase subunits from selected phages were used for phylogenetic analysis with *MEGA 6.0* [50].

## Results

### Genomic sequences of Bacillus phage isolates were almost identical within cluster

Single-contig assemblies were successfully generated from 39 independent isolates including SBP8a. Three clusters were generated from 38 single-contig isolates based on DNA sequence similarity including I48-like, Q8-like and Q11-like group, while SBP8a was separated into a single-isolate group. Cluster similarities were greater than 99.7% within alignable regions due to the isolation process from single soil sample. Isolates SBP8a and Q8 (QCM: Quartz-Crystal-Microbalance-spore adhering strain (unpublished data) had both been selected by adherence to spores and Q8 by simultaneous adherence to QCM electrode surfaces. Both were propagated by the same methods as all other isolates. However, the overall identity between SBP8a and Q8 was only 13%. Intriguingly, I48 and SBP8a shared approximately 90% identity over genomic sequence. The isolates I48 (157,912 bp; large-genome strain), Q8, 158,180 bp), Q11 (QCM, 26,005 bp; small-genome), and SBP8a (158,819 bp) were chosen as representative strains for genome similarity comparison (Table 1). Q11 possessed a one-sixth genome size in comparison to the other three strains. The sequence identity that Q11 shared with other representative isolates was less than 1.327% over 26,005 bp. More importantly, isolates within clusters were treated as biological repeats for subsequent analyses. The large number of repeats, up to 31 in I48-like cluster, allowed detailed interrogation of sequence characteristics in NGS data.

**Table 1:** Genome similarity among representative isolates

		Q8	SBP8a	Q11
I48	Query Coverage; Identities	1700/158180; 72%	151518/158819; 94%	70/26005; 81%
	Overall Identity	0.774%	89.679%	0.002%
Q8	Query Coverage; Identities		27614/158819; 75%	57/26005; 89%
	Overall Identity		13.040%	0.196%
SBP8a	Query Coverage; Identities			367/26005; 94%
	Overall Identity			1.327%

### Prediction of genomic termini by NGS data

The random start sites and orientation of contig sequences among 31 I48-like isolates was revealed after genome alignment (Additional file 1: Figure S1). The analysis of read alignment file also showed that some mappable reads that were located at the end of the contigs could map to both ends of the contig sequences. This suggested that these contigs were circularized. Nevertheless, the assembly programs could not resolve a circular genome; random cleavages on circularized contigs were produced, which resulted in linear contigs. Therefore, samples with reads that mapped to both contig ends were designated as circular in contig form in this study, while samples without those reads were linear contigs for subsequent analysis.

**Fig. 2:**
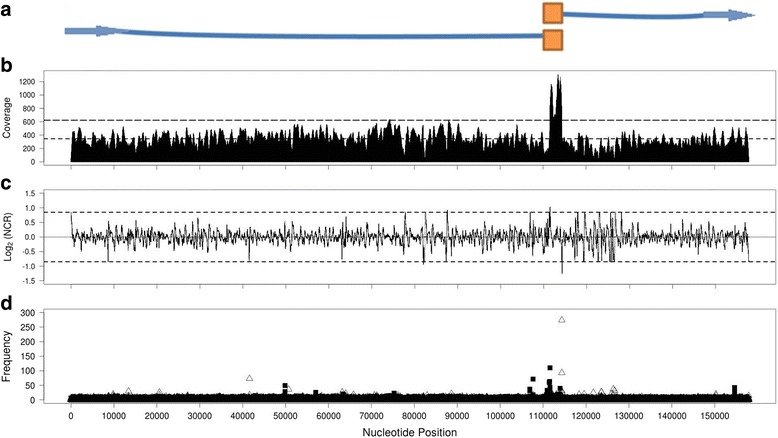
The map of coverage distribution, neighboring coverage ratio (NCR) and read edge frequencies of phage isolate I13. **a** An illustration of hypothetical genome configuration of I13 with terminal repeats. *Filled squares* indicate the direct terminal repeat of phage genome. **b** Coverage distribution over I13 sequence contig. The *lower dashed line* represents the average coverage of I13 sequencing reads. The *upper dashed line* represents the level of 1.8 times of average coverage. **c** Neighboring coverage ratio (NCR) over I13 sequence contig with window size = 100 bp. The *dashed lines* indicate the cut-off of 1.8 and reciprocal of 1.8 of NCR after base-2 logarithmic transformation [−0.848, 0.848]. **d** 5′ or 3′ read edge frequencies from I13 sequencing reads. *Filled black squares* indicate the frequencies of 5′ read edge positions. *Blank triangles* indicate the frequencies of 3′ read edge position

Coverage distributions of I48-like isolates featured regions that had evidently higher coverage than the rest of the contig (Additional file 1: Figures S2 and S3). We described these regions as “high coverage regions” in subsequent analyses. Two major characteristics were defined for the basis of algorithm in this study: (1) A regional highest NCR followed by a regional lowest NCR (Fig. 1a and b); (2) rank of read edge frequency calculated by read edge positions (Fig. 1c). Phage genomes with long direct repeats are known to feature terminal redundancy. Sequence coverage within terminal repeat region should be approximately 2-fold higher than non-terminal repeat regions since there are two copies of the repeat sequence template in one completely packaged genome. Based on the coverage distribution of I13 (Fig. 2b), an I48-like phage, we hypothesized that the high coverage region is where the direct terminal repeat (DTR) are located, and the boundaries of high coverage region are the physical ends of DTR (Fig. 2a). NCR and read edge frequencies were developed to demonstrate that NGS data has sufficient information to examine the positions of genomic termini in silico.

The purpose of calculating NCR was to locate boundaries of high coverage regions on a given contig. The NCR map of I13 (an I48-like phage), revealed one local highest NCR followed by one local lowest NCR around the edges of high coverage (Fig. 2c and d). The NCR selection process (as illustrated in Fig. 1a and b), suggested that nucleotide position 111,600 and 114,360 were boundaries of a repeat region (Table 2). This suggested an approximately 2760 bp high coverage region of the I13 contig. Instead of assuming a circular genome in nature, which is rarely found in *Myoviridae*, we suggested a linear genome with direct terminal repeats for I13 isolate based on the NCR results. The potential termini are likely the boundary positions of a high coverage region. The NCR analysis for every single-contig phage genome assembly was summarized in Additional file 1: Tables S2-S6.

**Table 2:** Summary of terminus prediction on selected isolates in this study and nine published phages

Phage	Sequencer	# reads	Coverage	Contig size	Contig form	^a^5′ hit	^b^5′ NCR	^c^5′ REP	^d^5′ REF	^e^3′ hit	^f^3′ NCR	^g^3′ REP	^h^3′ REF	5′ terminus flanking sequence	3′ terminus flanking sequence
Novel Phages															
SBP8a	*Roche/454*	38455	87.27	158794	Circular	**111794**	1.874	**111794**	117	**114616**	0.159	**114616**	238	5′-TCAGGTAGAA	AGAAAAACCT-3′
SBP8a	*MiSeq*	4662176	4670.87	158822	Circular	29634	1.889	30078	2434	32372	0.526	29983	2455	N/A	N/A
^i^I13	*PGM*	186893	345.92	157905	Circular	**111600**	2.036	**111610**	110	**114359**	0.42	**114359**	274	5′-AAACCGTATG	AGAAAAACCT-3′
I48	*MiSeq*	2975178	4042.41	157912	Circular	72862	1.819	73296	1332	74149	0.524	73201	1261	N/A	N/A
Q8	*PGM*	72799	115.53	158180	Circular	**156549**	68.497	**156549**	965	no hit	N/A	156836	114	5′-AGGTTTTGTG	N/A
Q11	*MiSeq*	2411528	15285.66	26005	Linear	no hit	N/A	15118	6717	no hit	N/A	15126	6090	N/A	N/A
Published phage with suggested packaging mechanism															
Direct Terminal Repeat															
Adelynn	*MiSeq*	250419	215.43	162356	Circular	**18032**	3.346	**18032**	261	**20724**	0.499	**20724**	171	5′-GGGTTTTTAT	CCGCCTACCC-3′
Nigalana	*Roche/454*	35451	98.06	160174	Circular	**6458**	2.296	**6458**	121	**9324**	0.542	**9324**	95	5′-AGGTTTTTCT	CGTTCTACCT-3′
Troll	*PGM*	32949	29.17	163019	Circular	no hit	N/A	62795	7	no hit	N/A	43962	9	N/A	N/A
Circular permutation															
Breeniome	*MiSeq*	138039	60.10	154434	Circular	no hit	N/A	26061	17	no hit	N/A	48207	19	N/A	N/A
Teardrop	*Roche/454*	9807	27.05	155389	Circular	no hit	N/A	10666	26	**11109**	0.497	**11109**	28	N/A	CCGCTCCGTT-3′
Zeenon	*PGM*	203150	179.03	155292	Circular	no hit	N/A	139104	23	no hit	N/A	10008	22	N/A	N/A
3′ overhangs															
Equemioh13	*MiSeq*	150088	394.34	53042	Circular	**40880**	4.803	**40880**	276	no hit	N/A	41030	191	5′-TGCGGCCGCC	N/A
Zetzy	*Roche/454*	9157	80.92	48463	Linear	**34529**	3.321	**34529**	116	no hit	N/A	34586	70	5′-CCTGTGCGCC	N/A
Lilith	*PGM*	215716	668.86	50827	Circular	no hit	N/A	5180	89	no hit	N/A	3846	113	N/A	N/A

The numbers in bold font indicate the significant hits of potential termini

^a^5′ hit: nucleotide position of significant NCR hit at 5′ boundary of high coverage region

^b^5′ NCR: the ratio of 5′ significant NCR hit

^c^5′ REP: 5′ read edge position with highest frequency

^d^5′ REF: read edge frequency at 5′ REP

^e^3′ hit: nucleotide position of significant NCR hit at 3′ boundary of high coverage region

^f^3′ NCR: the ratio of 3′ significant NCR hit

^g^3′ REP: 3′ read edge position with highest frequency

^h^3′ REF: read edge frequency at 3′ REP

^i^I13 is one of I48-like isolate

It is crucial to have a second and independent method in order to increase the robustness of terminal prediction. Read edge frequency was used as another trait of potential termini predicted by NGS data. Library preparations for *PGM* and *MiSeq* sequencing have genome fragmentation/shotgun steps. Assuming that every nucleotide position has an equal probability to be fragmented during shotgun library preparation, we hypothesized that the fragments that contain the first base pair or the last base pair of a linear genome should have the highest frequency in a genome fragmentation pool. Assuming a non-biased amplification and sampling for sequencing, high read edge frequencies at genome termini are expected. The read mapping result of isolate I13 indicated that 110 reads had their 5′ end aligned at nucleotide position 111,610, while the 3′ end position occurred 274 times at position 114,359 bp (Table 2). These were the highest 5′ and 3′ read edge frequencies across the whole contig, which were considered as predicted termini for I13 (Fig. 2d). Furthermore, the positions with the highest read edge frequency in I13 were proximal to the predicted terminal positions by NCR (Table 2), located within the terminal windows.

Most terminus prediction results for I48-like phages from *PGM* featured identical genomic terminal sequence 5′- AGGTTTTTCT while the 3′ terminus was CATACGGTTT-3′. I48-like isolates appeared to have a linear genome about 158 kb in length with an additional 2750 bp DTR. NCR data from SBP8a showed potential terminus at position 111,794 bp and 114,616 bp, which also suggested a linear genome with DTR based on *Roche/454* sequencing data. The highest frequency of 5′ and 3′ read edge positions occurred at the same positions in NCR prediction of SBP8a (Table 2). NCR and read edge frequencies failed to suggest a definite terminal position for SBP8a from *MiSeq* sequencing data (Additional file 1: Table S6), although a relative high coverage region was observed (Additional file 1: Figure S4). Biased nucleotide frequency at the 5′ end of *MiSeq* reads was identified by FastQC. The sequence content per base of SBP8a paired-end reads (SBP8a.R1 and SBP8a.R2) showed that the nucleotide frequency of A and T were 20% more than that of C and G at the first 10 bp of *MiSeq* reads (Additional file 1: Figure S7). This trend has been attributed to the sequence selection bias of transposase during the fragmentation process in the *Nextera* library preparation in previous study [51, 52]. In order to test whether the sequence pattern exists at both ends of the *MiSeq* reads, the first 20 bp at 5′ end and 20 bp upstream of *Nextera* adapter CTGTCTCTTATA at 3′ end from every read were collected for sequence pattern analysis (Additional file 1: Figure S7 and S8). The sequence logo showed highly conserved sequence 12 bp from both edge of reads. Furthermore, the sequence patterns were almost symmetric at both read edges flanking by the *Nextera* adapter. While the *MiSeq* reads showed strong sequence bias, the *PGM* reads did not possess a sequence pattern at read ends (Additional file 1: Figure S9). The overall height on sequence logos generated by *PGM* reads was relatively smaller than that of *MiSeq* reads across the 20-bp screening regions. The biased selection of read edge position by *Nextera* transposome misrepresented the read edge frequency, hence gave rise to negative result of read edge frequency for NGS-based terminus prediction.

For NGS data analyses of Q8-like isolates, one common terminus was found with flanking sequence 5′-AGGTTTTTGTG on Q2, Q8 and Q10 (Additional file 1: Table S4), which is close to the 5′ boundary of high coverage region. There was no significant hit at a 3′ boundary of high coverage region in NCR analysis. None of the Q11-like isolates had significant NCR hit that suggested terminal repeats in our data (Additional file 1: Table S5). The coverage distributions of Q11-like isolates showed fluctuations without abrupt high coverage region across the contig (Additional file 1: Figure S6). Furthermore, there was no sequencing read that was able to align across both ends of corresponding contigs in isolates of the Q11-like group, in which case these contigs appeared to be linear. Under the hypothesis that the sequence fragments containing the physical ends of a linear genome have the highest frequency in a genome fragmentation pool, this result suggested that the contig sequences of Q11-like isolates were incomplete from assemblies. Nevertheless, the negative result from NGS analysis might attribute to other characteristics of a phage genome configuration that is not accessible by sequencing data. Proposed solution along with current understanding of phage genome packaging mechanisms were described in discussion section.

### Terminal sequence validation from primer walking

*Taq* polymerase extends one non-templated adenine after the last base of a newly synthesized strand when the elongation reaches the terminus of template, which is commonly seen on chromatograms from Sanger sequencing technology [53]. ‘Primer walking’ takes advantage of this feature to design primers that walk toward genome termini until the observation of non-templated adenine from Sanger sequencing. Therefore, primer walking help validate the last base pair of a genome where termini were predicted in silico from NCR and read edge frequencies. An example of primer design and Sanger sequencing chromatogram of SBP8a, a proposed linear genome with direct terminal repeats, showed the observation of non-templated adenine in Additional file 1: Figure S10. When a primer is designed within a terminal repeat region and elongated toward the terminus using a template genome with direct terminal repeat, the chromatogram is expected to have a ‘mixture’ signal from two templates. The elongation of DNA synthesis would be terminated at the physical end of one copy of template DNA and add a non-templated adenine after the last base pair of newly synthesized DNA, while another copy of the template allows the elongation of sequence synthesis toward non-redundant region. As a result, the signal intensity of chromatography after the physical ends would decrease by one-half on a linear DNA template with terminal repeat, along with a non-templated adenine generated by *Taq* polymerase. In contrast, primer walking on a template without any genome redundancy would expect a complete termination of sequencing after the non-templated adenine is observed. However, primer walking cannot identify either 5′ or 3′ overhang without a terminal ligation step before direct sequencing.

Our primers were designed from sequence approximately 150–250 bp upstream of predicted termini (Additional file 1: Table S9). Primer walking was used to confirm the genome termini for isolates and to validate the termini predictions from NGS data (Table 3). By comparing predictions from NGS data to primer walking methods, the genome terminus position for SBP8a phage matched most closely, having only a two base-pair difference on 5′ terminus and an exact match on 3′ terminus. The 5′ termini of I13 and I22 were 73 bp upstream of the ‘coverage-predicted’ terminal sequences, while 3′ termini had zero to three base pair differences from predicted positions. Notably, the predicted 3′ terminus of Q8-like isolates used for primer walking was determined based on common positions derived from the top 10 ranking 3′ read edge frequencies that the three Q8-like sequences shared (TATTTTTCGA-3′), rather than from the highest frequency termini among read edge positions (Additional file 1: Table S4). Location of Q8-like strain physical ends was resolved by primer walking from 360 bp upstream of the predicted terminus position of Q8 isolates. The precise Q8 terminal position was revealed at position 5099, which is 74 bp downstream of the NGS predicted position at position 5025 on Q8 assembled contig. The genome configuration of Q8 was therefore determined as a linear genome with 6731 bp DTR. Interestingly, the terminal sequence starts with 5′-AGGTTTT…, which is shared among SBP8a (AGAAAAACCT-3′), I13 (AGAAAAACCT-3′), I22 (AGAAAAACCT-3′), Q8 (5′- AGGTTTTGTG) and Q10 (5′- AGGTTTTGTG). This identical terminal sequence suggests that the sequence-specific terminus is likely to be the terminase recognition site or cleavage site on concatemeric DNA where the packaging complex initiates DNA packaging. The data of primer walking supported that SBP8a, I48-like and Q8-like phages had DTRs with exact lengths and a position-specific cleavage sites. Although the terminus was thoroughly defined in this study, the relationship between the specific terminus and large terminase protein needs further investigation.

**Table 3:** Comparison of terminal position between the prediction from NGS data and identification from primer walking method

Terminus predicted by NGS data					Terminus identified by primer walking sequencing						
Name	Sequencer	Contig size	^a^5′ ter.	^b^3′ ter.	5′ ter.	^c^distance	5′ terminus flanking sequence	3′ ter.	distance	3′ terminus flanking sequence	length of DTR
SBP8a	*Roche/454*	158794	111794	114616	111796	+2	5′-AGGTAGAACG	114616	0	AGAAAAACCT-3′	2821
^d^I13	*PGM*	157905	111610	114359	111537	−73	5′- AGGTAGAACG	114359	0	AGAAAAACCT-3′	2823
^d^I22	*PGM*	157889	106421	109166	106348	−73	5′- AGGTAGAACG	109169	+3	AGAAAAACCT-3′	2822
Q8	*PGM*	158180	156549	^g^5025	156549	0	5′- AGGTTTTGTG	5099	+74	GGGTCTACCC-3′	6731
^e^Q10	*PGM*	158174	86402	^g^93056	86402	0	5′- AGGTTTTGTG	93130	+74	GGGTCTACCC-3′	6729
Q11	*MiSeq*	26005	N/A	N/A	^h^-9	N/A	5′ AAAATGTAAA	^h^ + 43	N/A	ATATACATTT-3′	N/A
^f^I46	*MiSeq*	24896	N/A	N/A	^h^-740	N/A	5′ AAAATGTAAA	^h^ + 421	N/A	ATATACATTT-3′	N/A

^a^5′ ter.: nucleotide coordinate of 5′ terminus

^b^3′ ter.: nucleotide coordinate of 3′ terminus

^c^distance: position difference to prediction

^d^I13 and I22 are I48-like isolates

^e^Q10 is one of Q8-like isolate

^f^I46 is one of Q11-like isolate

^g^Location of Q8-like stain physical ends was assisted by designing primers from several hundred bases upstream of the common predicted hits

^h^The position outside of contig sequence was calculated by the relative position of coordinates of contig sequence from NGS data. Minus coordinate represents 5′ upstream of first bp of contig; Plus coordinate represents 3′ downstream of last bp of contig

For phage Q11, we designed primers at approximately 150 bp upstream of contig ends and performed the Sanger sequencing to verify complete sequencing from NGS data. Surprisingly, primer-walking revealed that Q11-like phage genomes had two position-specific cleavage sites outside of the contig. The non-templated ‘A’ call occurred at 9 bp upstream of the first nucleotide and 43 bp downstream of last nucleotide of Q11 contig. These sequences were the fragments that were not resolved from genome assembly. The possibility of insufficient sequencing data is less likely since the average coverage among Q11-like phages is between 2077.66 and 12345.86, which is relatively higher than other sequenced phages in this study. Rather, it is likely the result of masked terminus due to the impure sample with covalently bound terminal proteins.

### Calibration of terminus prediction with published NGS data from nine phages

Our terminus predicting method performed fairly accurate prediction for novel phage isolates. We additionally examined the prediction method on published NGS data from nine phages with characterized genome termini in the database. Three known types of phage genomic termini were included in this calibration: DTRs, circularly permuted genomes and 3′ overhang termini. Three different sequencing platforms for each type of genome terminus allowed us to investigate whether the sequencing platform has an effect on terminus prediction.

Table 2 summarized terminus prediction of nine phage NGS data in this calibration. The prediction suggested that *Bacillus* phage Adelynn and Nigalana have 2693 bp and 2867 bp DTR, respectively. The predicted lengths of terminal repeats were exactly the same size as phageDB reported (Additional file 1: Table S8). Intriguingly, the 5′ terminus of Adelynn (5′- GGGTTTTTAT) and Nigalana (5′- AGGTTTTTCT) are mostly similar to the initiating terminus on SBP8a, I48-like and Q8-like phages (5′-AGGTTTT). Neither our method nor phageDB report identified the signal of terminus of Troll phage though there was a high coverage region indicated between 85 kb and 88 kb (Additional file 1: Figure S11). Phages characterized as circularly permuted genomes tend to have linear contigs without a consistent terminus among virions. The contigs of Breeniome, Teardrop and Zeenon were characterized as circular due to the existence of reads across both contig ends. Terminus prediction attempts on Breeniome, Teardrop and Zeenon (characterized circularly permutated phages) failed to identify a distinguishable terminus except for the 3′ end of phage Teardrop. This terminus on Teardrop may be the first unit-length genome that was cleaved from a concatemer, which had a consistent cleavage site near the *pac* site [31]. Among the three 3′ overhang phage genomes, our prediction method identified a potential terminus on Equemioh13 that was immediately adjacent to the reported positions of terminal sequence in The Actinobacteriophage Database (http://phagesdb.org/) (Additional file 1: Figure S12, Table S8). Zetzy showed to be linear after assembly due to the lack of reads mapped across contig ends, which implies that the ends of Zetzy contig are the genomic termini. Nevertheless, Zetzy was predicted as a linear genome with a terminus at position 34,529 bp by the prediction method. However, Zetzy had aa 3′ overhang terminus (5′-CGGGTGGTAA) reported on the database (Table 2, Additional file 1: Table S8). This is a case where terminus could not be informed due to the incomplete information in the NGS data. Lilith phage genome had no clear high coverage region sufficient to surpass our criteria for terminus prediction; therefore a potential terminal sequence was not determined.

### Phylogenetic clustering by terminase large subunit and implications for types of packaging mechanisms

**Fig. 3:**
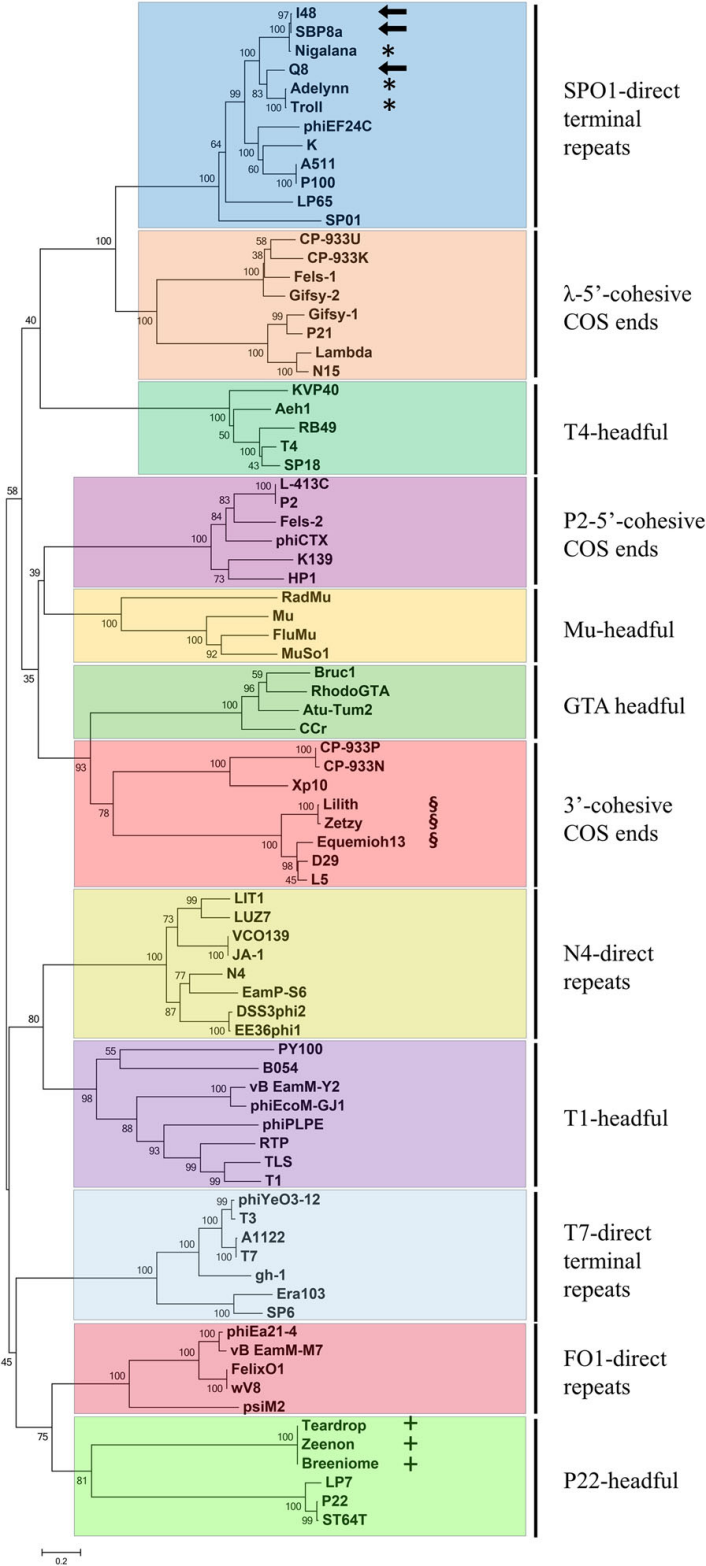
Maximum Likelihood phylogeny of large terminase amino acid sequences. The alignment of protein sequences was generated by ClustalW2 [65]. The phylogeny was reconstructed using Maximum Likelihood method based on the Poisson correction model. Numbers next to internal nodes indicate the bootstrap value divided by trials size of 1000. Names of phages were illustrated at the tip of the phylogeny. The root of the phylogeny was arbitrarily chosen for visualization purpose. *Arrows*: three novel *Bacillus* phages including SBP8a, I48 and Q8. *, +, &: nine phages with suggested types of genome terminus

The annotated amino acid sequences of TerL from genomes of I48, Q8, SBP8a and nine prediction validating phages were identified based on ORF prediction and homologous sequence search with BLASTP as well as HHblits. These twelve TerL were then aligned with 69 known large terminase protein sequences of phages, covering as many packaging strategies as are available from current literature (Fig. 3). Neither significant E-value (< ${10}^{-10}$ in BLASTP) nor probability (>80% in HHblits) for terminase large subunit was found on Q11 genome, which indicated that Q11 genome does not have a putative TerL gene. I48, Q8 and SBP8a clustered with SPO1-related phages, inferring that those genomes have long DTRs. This result again confirmed the terminus prediction method from NGS data for I48, Q8 and SBP8a. Adelynn, Nigalana and Troll that are known to have direct terminal repeats were also clustered in the SPO1-like clade. Equimioh13, Zetzy and Lilith were closely related to L5 and D29 that are known to have 3′ overhangs at genome terminus. Breeniome, Zeenon and Teardrop were clustered with P22-like phages, which are circularly permuted genomes.

## Discussion

Contig circularity was found in majority of NGS sequenced phages analyzed in this study (35 out of 39 single-contig novel isolates; eight out of nine published phages). According to current understanding of tailed phage genome configuration, most of phage genome sequences feature at least a portion of sequence redundancy in the virion genome. Mu-like and phi29-like phages with unique DNA packaging process are the exceptions. To be more specific, read mapping results of tailed phages with 5′ or 3′ protruding ends reveal an overlapping region mapped at cohesive sequences. This results in a circular map in assembler programs, which return sequences that start from an arbitrary location. The prediction method developed in this study has shown to resolve the genome configuration of sticky-end phages such as Equemioh13. A completely assembled contig of DTR genome, as we demonstrated in the study, is circularized. Using NCR and read edge frequencies, the absolute or proximal coordinates of DTR can be identified. The sequencing data of a circularly permuted phage would of course form a circular-like map during assembly since headful packaged virions feature diverse termini. However, a study that analyzed high-coverage reads of T4-like phage IME08 in NGS data revealed that the T4-like phage might have a sequence-specific cleavage on one terminus of the genome [33]. Circularity of phage contigs is necessary to assure acquisition of a complete tailed phage genome, as well as to address terminus prediction using methods in this study or similar studies. We note that no phages with 5′ cohesive ends were examined in the study. Additionally, it is insufficient to identify the precise overhang terminus using Sanger sequencing without the comparison of terminal ligation treatment. Further work is required to determine whether contig circularity is common in genome assembly for most of tailed phages through NGS data, especially for phages with 5′ or 3′ overhang ends.

An unexpected factor that disfavored the NGS-based terminus prediction was found on *Nextera* transposon-based library preparation kit for *MiSeq* sequence production. Our data suggested a biased sequence selection at both read edges (Additional file 1: Figure S7 and S8). This bias does not have evident effect on de novo genome assembly, but it caused misrepresentation of the distribution of read edge positions that was crucial for the terminus prediction method developed in this study. It is known that Tn5 Transposase recognizes inverted repeats on both ends of transposable element (reviewed in [54]). The recognition site of Tn5 has been reported as a 19-bp end sequence based on several genetic analyses [55–60]. As for the insertion event, Green et al. demonstrated insertion bias of Tn5 by a fosmid library screening method [52]. It is hypothesized that the Tn5-mediated transposition favors sequences that contain partial, if not complete, homology to the 19-bp recognition sequence. *Nextera* preserves this property on modified transposomes during DNA library preparation, which results in read selection bias. Schirmer and colleagues reported the transposome sequence bias in a systematic profiling of Illumina sequencing [61]. The analysis of read end conservation on *MiSeq* reads agreed with previous conclusions that *Nextera*-derived Illumina sequence favored a sequence pattern flanking the insertion of the *Nextera* adapter. The sequence motif is roughly 12 bp from the breakpoint of the *Nextera* adapter. The transposition bias is a crucial disadvantage for the NGS-based terminus prediction in terms of read edge frequency. Note that there were no biased sequences shared by both read ends from the Illumina-generated runs for Adelynn, Breeniome and Equemioh13 (Additional file 1: Figure S13). The fragmentation step of these phages was implemented by dsDNA shearase (Zymo Research) in Hatfull’s lab. It indicates that the generation of the biased-ended reads in our isolates was due to library preparation but not Illumina sequencing platform. It is suggested that a standard FastQC followed by sequence pattern search should be performed before the implementation of terminus prediction.

A linear genome was observed in Q11-like phage isolates. The Q11 terminal sequences were discovered by primer walking but not with the terminus prediction method developed in this study. Previous studies have shown that phi-29 like phages have terminal proteins at the genome terminus that might interrupt genome sequencing [62, 63]. This could be tested for Q11-like genomes by using an additional protease K treatment step during the DNA extraction in future experiments. It is also possible that the terminal sequences tend to form secondary structures that are difficult to address during library preparation or sequencing.

The reconstructed phylogeny based on amino acid sequence of phage terminase large subunit produced clusters associated with types of genome terminus. This result supported the prediction that the three novel phages (SBP8a, I48 and Q8) have linear genomes with direct terminal repeats. While the amino acid sequences of TerL were widely used to correlate the type of DNA packaging with genome configuration, genetic recombination or horizontal gene transfer could change the DNA sequence of phage TerL gene over evolutionary time and could perturb the inference of ‘phage clustering’ by phylogenetic analysis [64]. Therefore, DNA packaging mechanisms should be determined with caution by further experimental analysis rather than TerL phylogeny alone.

## Conclusions

In this study, we demonstrated that the contig circularity is an important feature to acquire complete genome sequences from most of the tailed phage NGS data. Phage genome terminus prediction based on NGS data is an efficient method to identify the proximity of terminal sequences. The identification of phage genome termini allows insight into potential DNA packaging mechanisms. Primers within redundant regions can be used to confirm terminal sequences via Sanger sequencing. This work suggests that sequence coverage provided by NGS data is sufficient to identify the terminal sequences of de novo linear phage genomes when single-contig and circularized assemblies are generated.

## Additional file

Additional file 1: Supplemental material and method. (DOCX 10269 kb)

## Abbreviations

DTR: Direct terminal repeat
NCR: Neighboring coverage ratios
NGS: Next-generation sequencing
QCM: Quartz crystal microbalance
Ter: DNA terminase
TerL: Large terminase subunit of DNA terminase
TerS: Small terminase subunit of DNA terminase
