# Supplementary material for: Predicting genome terminus sequences of Bacillus cereus-group bacteriophage using next generation sequencing data
Source: BMC Genomics. 2017 May 4;18:350. doi: 10.1186/s12864-017-3744-0 (PMC5418689; doi:10.1186/s12864-017-3744-0)
Supplement: Additional file 1: — Supplemental material and method. (DOCX 10269 kb) [file 12864_2017_3744_MOESM1_ESM.docx]

**Additional file**

**Predicting Genome Terminus Sequences of *Bacillus cereus*-group Bacteriophage using Next Generation Sequencing data**

Cheng-Han Chung, Michael H. Walter, Luobin Yang, Shu-Chuan (Grace) Chen, Vern Winston, Michael A. Thomas

**TABLE OF CONTENTS**

**Supplemental material and method**

**Figure S1**. A genome alignment of 31 I48-like isolates

**Figure S2**. Coverage distribution of 11 I48-like isolates sequenced by *Ion Torrent PGM*

**Figure S3**. Coverage distribution of 20 I48-like isolates sequenced by *MiSeq* genome sequencer

**Figure S4**. Coverage distribution of SBP8a isolates sequenced by *Roche/454 or MiSeq* genome sequencer

**Figure S5**. Coverage distribution of three Q8-like isolates sequenced by *PGM* genome sequencer

**Figure S6**. Coverage distribution of four Q11-like isolates sequenced by *MiSeq* genome sequencer

**Figure S7.** The sequence content across all bases and sequence logo of first 20bp or last 20 of SBP8a paired-end reads SBP8a.R1 (A) and SBP8a.R2 (B).

**Figure S8.** Sequence logo of I48-like phages sequenced by *MiSeq*.

**Figure S9.** Sequence logo of I48-like phages sequenced by *PGM*.

**Figure S10**. Coverage distribution of nine previously sequenced phages by *MiSeq*, *Roche/454* or *PGM* genome sequencer

**Figure S11**. Coverage distribution of nine previously sequenced phages by *MiSeq*, *Roche/454* or *PGM* genome sequencer

**Figure S12**. Coverage distribution of Equemioh13 from contig position 40,860 to 40,889

**Figure S13.** Sequence logo of nine previously sequenced phages by MiSeq, Roche/454 or PGM genome sequencer

**Table S1.** Summary of 23 phage genome assemblies from sequences by *Ion PGM*.

**Table S2.** Summary of 26 phage genome assemblies from sequences by *MiSeq* paired-end sequencing.

**Table S3**. Genome terminus prediction of 31 I48-like isolates using NGS data

**Table S4**. Genome terminus prediction of 3 Q8-like isolates using NGS data

**Table S5**. Genome terminus prediction of 4 Q11-like isolates using NGS data

**Table S6**. Genome terminus prediction of SPB8a isolate using NGS data

**Table S7**. Genome terminus prediction of 9 published isolates using NGS data

**Table S8.** A comparison of genome end characterization between published data on phageDB and predicted result by Terminus package.

**Table S9.** Primer sequences in primer walking for validating physical ends of phage genomes

**Materials and Methods**

*Genome assembly and basic characterization of Bacillus phage isolates*

Forty eight novel isolates were sequenced by *PGM* or *MiSeq,* and Spore-binding phage 8a (SBP8a) was sequenced by *MiSeq*. SBP8a has been previously characterized as *Myoviridae* and characterized to have *B. anthracis* spore-binding activity ([1](#_ENREF_1)). The average lengths of reads generated were 292.29bp by *PGM* and 228.12bp by *MiSeq* (Table S1 and S2). Expected per-run yields based on manufacturers’ specifications were 0.3-1.0 Gb for *PGM* *ion 316 chip* and 13.2-15 Gb for *MiSeq* *Reagent Kit v3*. Total yields in base pairs were 1.06 Gb by *PGM* and 12.59 Gb by *MiSeq* in a single flow cell.

Single-contig assemblies were successfully generated from 39 isolates. The sequence lengths of these 39 isolates fell into two major size categories: 35 isolates had approximately 158 kb genomes and four isolates had up to 26 kb genomes. For the latter four isolates, the contig lengths of I3, I17, I46 and Q11 ranged from 21,717 to 26,005 bp. These contigs all contained identical sequence within aligned regions, except I17, after multiple sequence alignment by ClustalOmega (Table S3) ([2](#_ENREF_2)). Three of the larger genome isolates Q2, Q8 and Q10 had fewer than three nucleotide differences in pairwise comparisons (Table S4). For the remaining 31 larger genome isolates with contig lengths of about 158 kb, pairwise alignments showed that the number of nucleotide differences between any two isolates were less than eight after correction of indels (insertion/deletion) in homopolymeric regions between *PGM* and *MiSeq* sequencer as described in the methods section (Table S5).

The assemblies of six *PGM*-sequenced genomes (Isolate I2B, I6, I15, I37, Q11 and Q1) failed to generate single contigs by *Newbler* assembler. These six genomes had more than 280 contigs after assembly and total contig lengths were too large for potential phage genomes. The second largest assembled contigs from each of the six isolates were subjected to BLAST searches against non-redundant nucleotide database in NCBI. The significant hits included 23S rRNA, plasmid, and intergenic region of various bacteria strains, suggesting that those samples were contaminated by host or environmental bacteria DNA. Three assemblies (I33, I35 and Q5) had two major contigs instead of one, each of which was over 1,000 bp in length. They featured gaps of 12 to 167 bp between the two contigs after mapping I33 and I35 contigs against I48, and Q5 against Q8. I50 yielded two major contigs which appeared to represent partial genomes of large-genome phages and small-genome phages. To avoid this ambiguity and incorrect assemblies, these ten isolates (I2B, I6, I15, I37, Q11, Q1, I33, I35, Q5 and I50) were excluded from subsequent analyses.

Among the remaining 39 single-contig genome assemblies, the average coverage of the 25 *MiSeq* contigs was 1927.21, which was 7.02 times higher than that of the 14 *PGM* contigs (274.57). Five out of six incomplete genome assemblies were sequenced by *PGM*. This suggests that the high coverage of assemblies from the *MiSeq* system increases chances to resolve complete viral genomes.

**Reference:**

1. **Walter MH, Baker DD.** 2003. Three Bacillus anthracis bacteriophages from topsoil. Curr Microbiol **47:**55-58.

2. **Larkin MA, Blackshields G, Brown NP, Chenna R, McGettigan PA, McWilliam H, Valentin F, Wallace IM, Wilm A, Lopez R, Thompson JD, Gibson TJ, Higgins DG.** 2007. Clustal W and Clustal X version 2.0. Bioinformatics **23:**2947-2948.


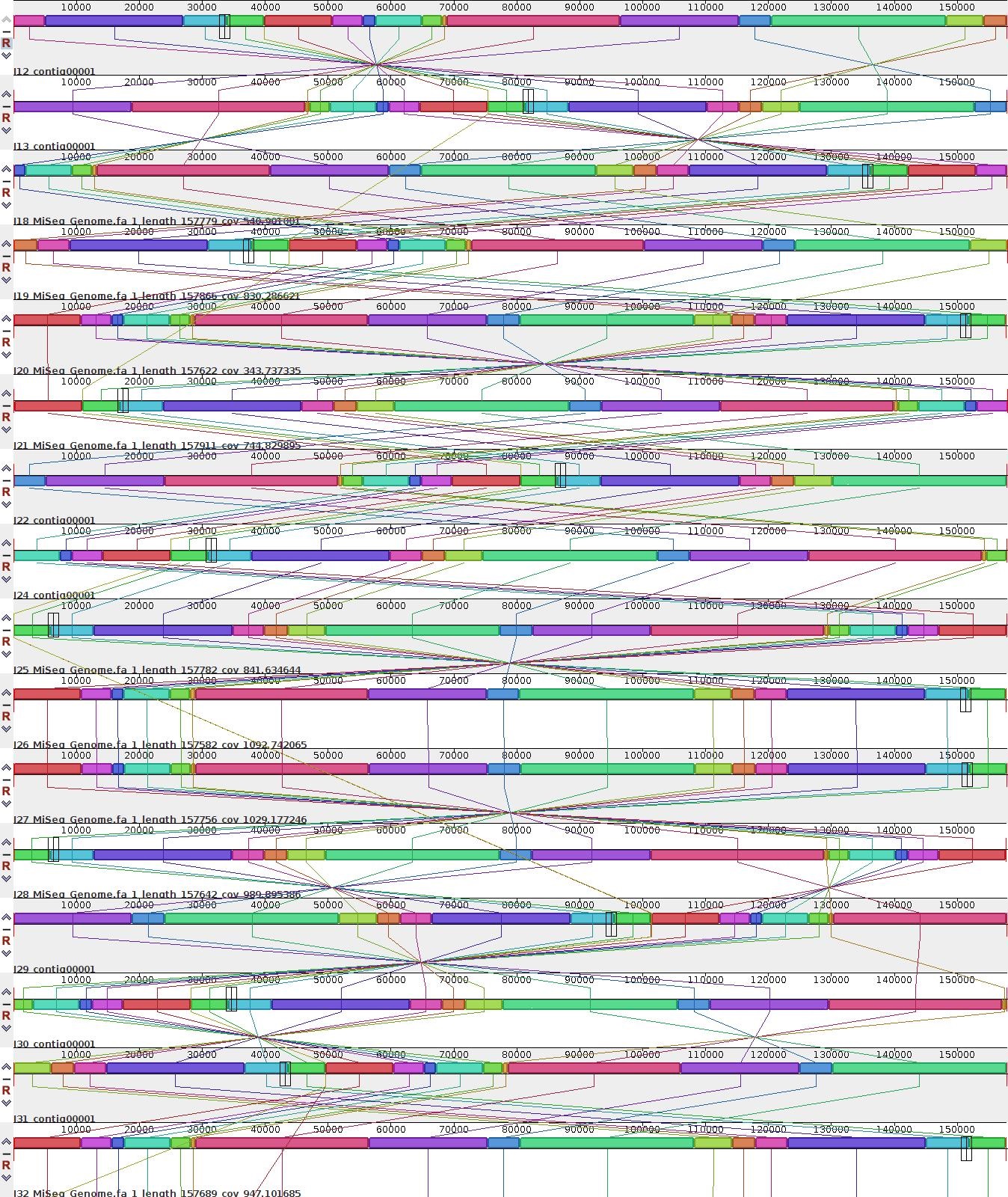


Figure S1 (Continue on next page)


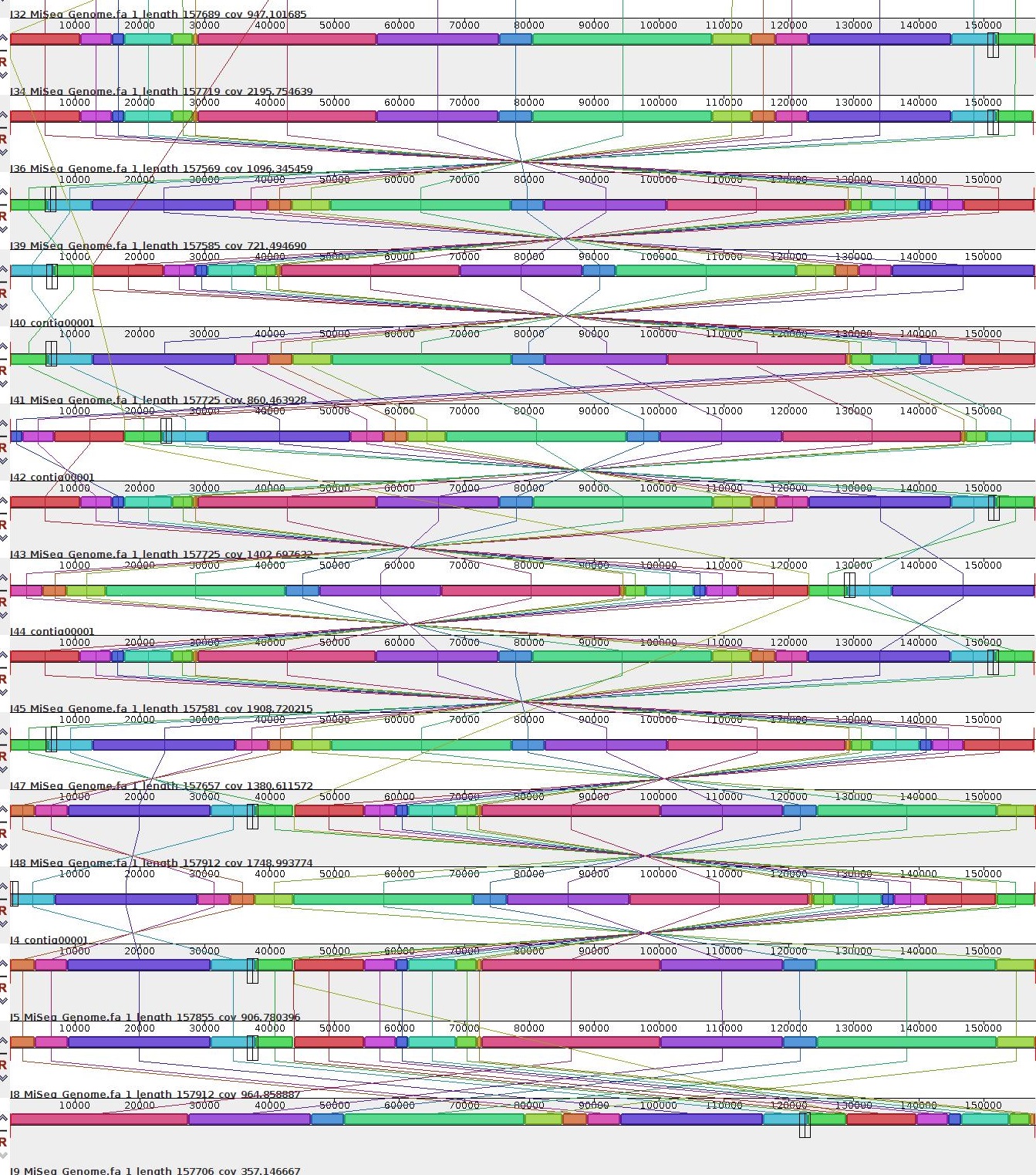


**Figure S1. A genome alignment of 31 I48-like isolates.** Whole genome alignment using Mauve (Darling et al., 2004) generated 16 Locally Collinear Blocks (LCB) conserved among isolates. A LCB with the same color showed homologous sequence among horizontal genome.


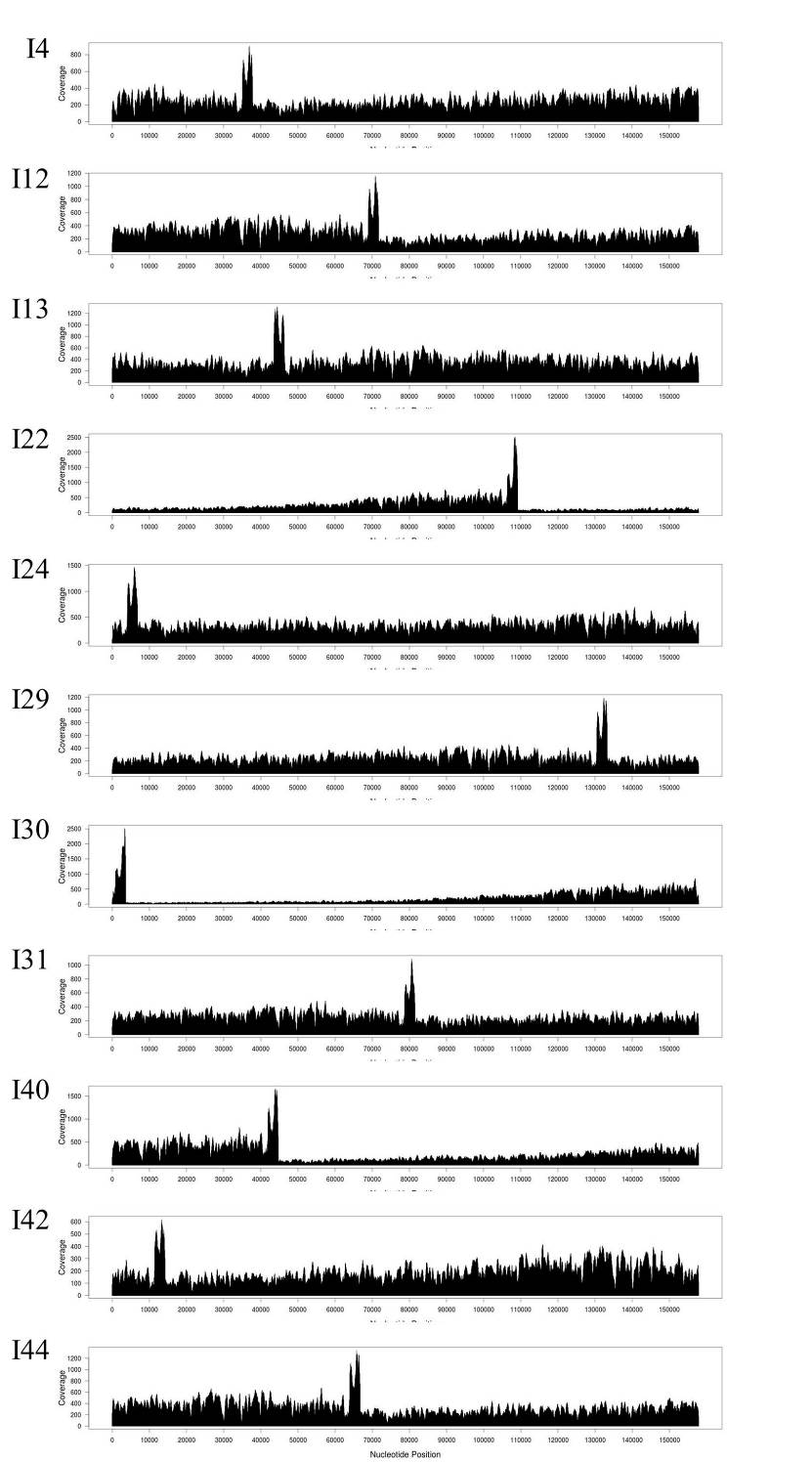


**Figure S2. Coverage distribution of 11 I48-like isolates sequenced by *Ion Torrent PGM*.** X-axis represents the nucleotide position of assembled contig; y-axis represents the coverage on the corresponding position.


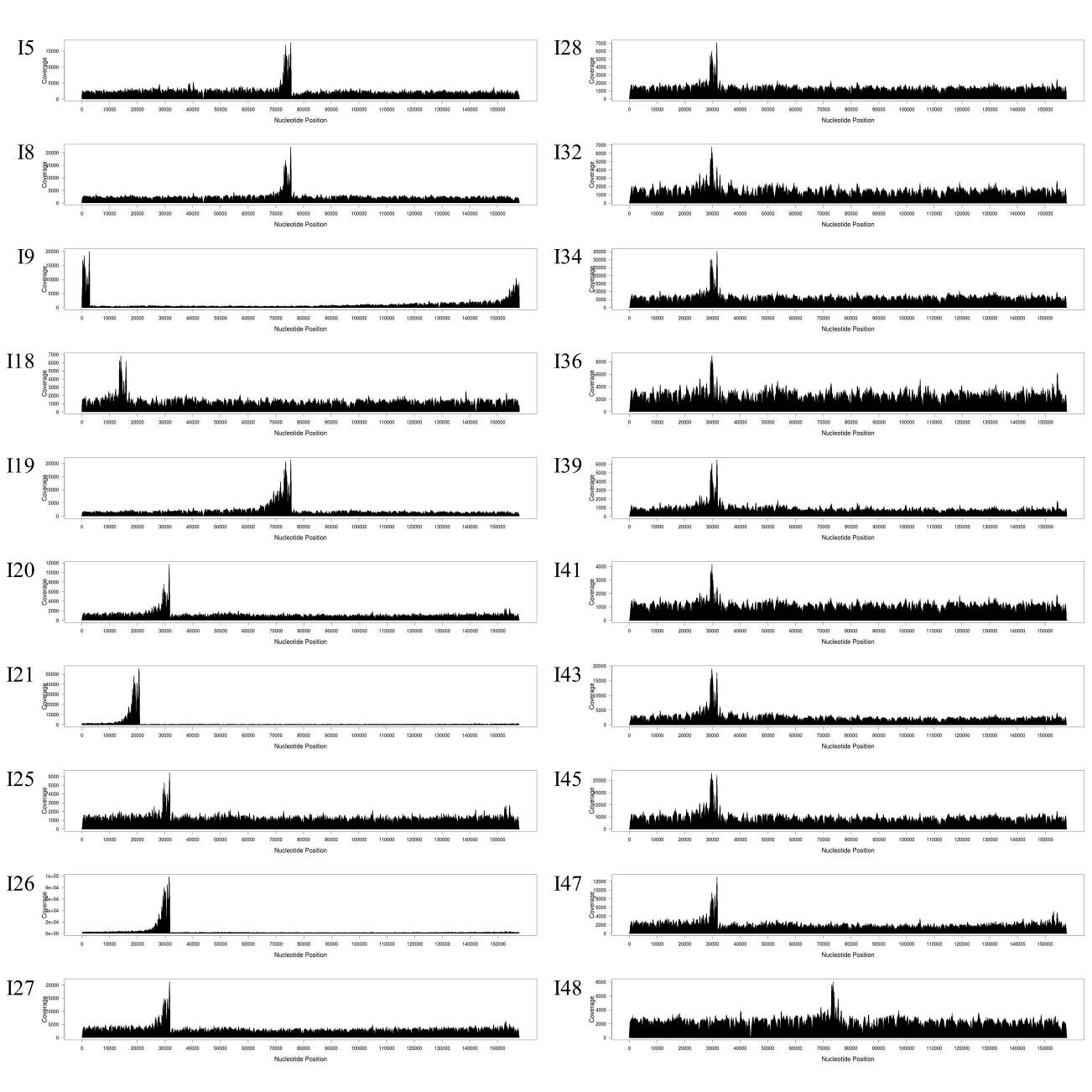


**Figure S3. Coverage distribution of 20 I48-like isolates sequenced by *MiSeq* genome sequencer.** X-axis represents the nucleotide position of assembled contig; y-axis represents the coverage on the corresponding position.


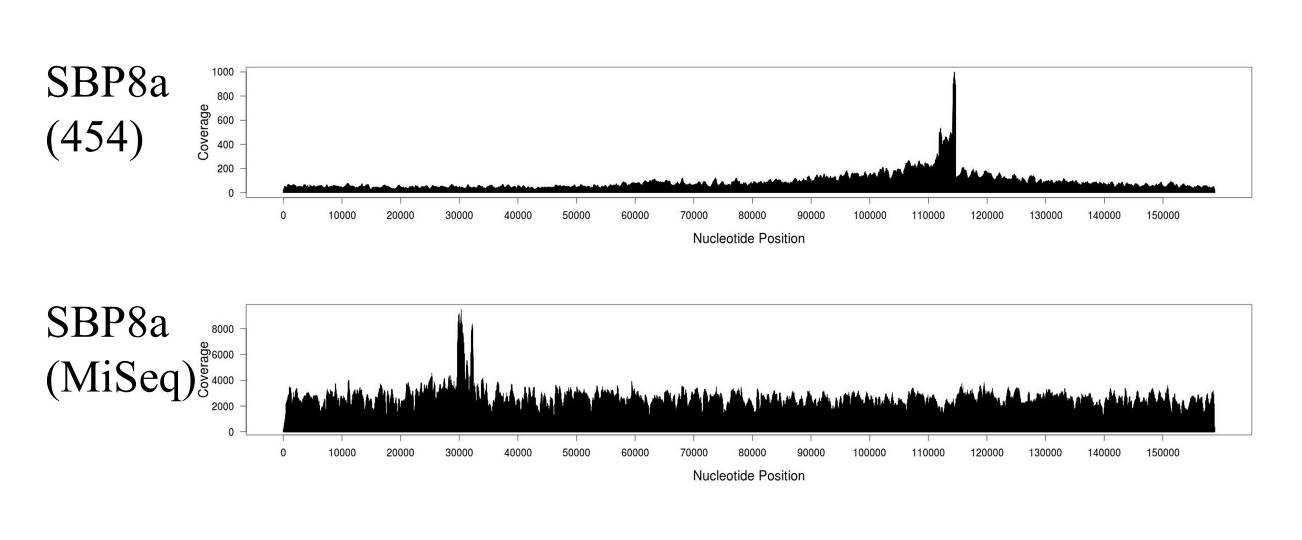


**Figure S4. Coverage distribution of SBP8a isolates sequenced by *Roche/454* or *MiSeq* genome sequencer.** X-axis represents the nucleotide position of assembled contig; y-axis represents the coverage on the corresponding position.


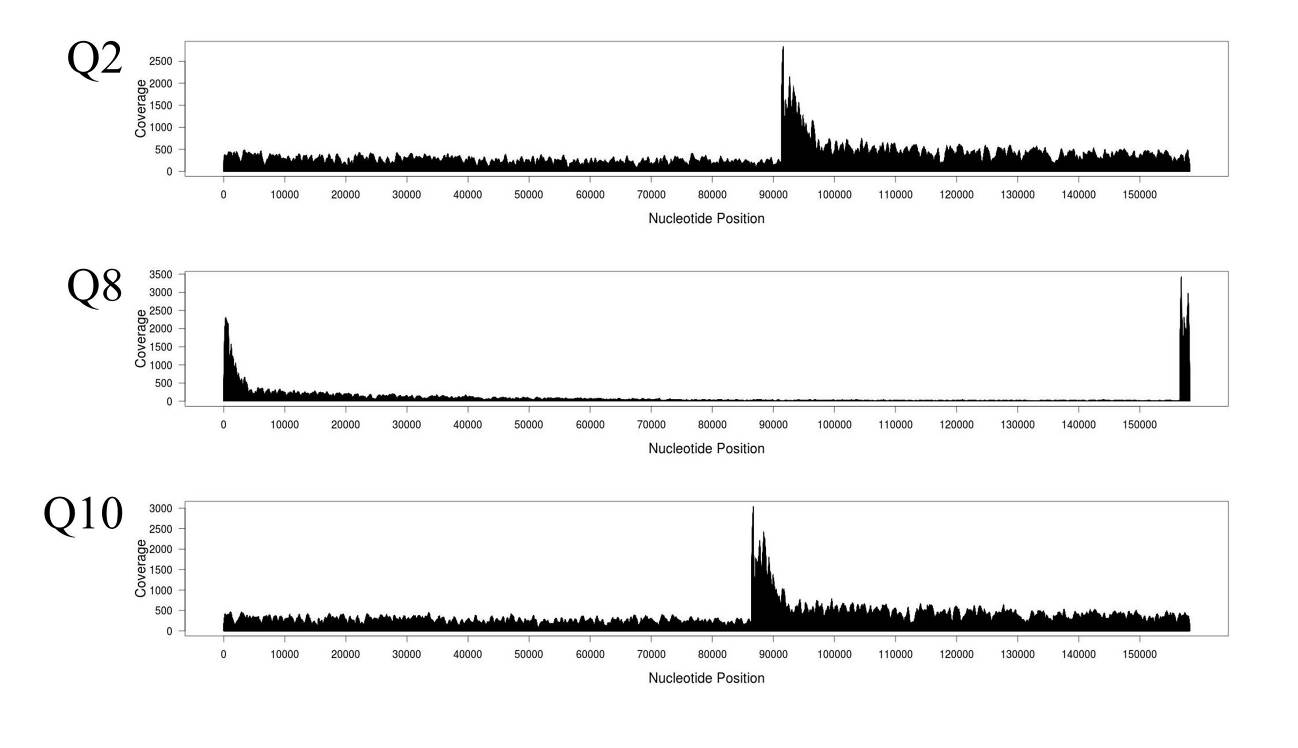


**Figure S5. Coverage distribution of three Q8-like isolates sequenced by *PGM* genome sequencer.** X-axis represents the nucleotide position of assembled contig; y-axis represents the coverage on the corresponding position.


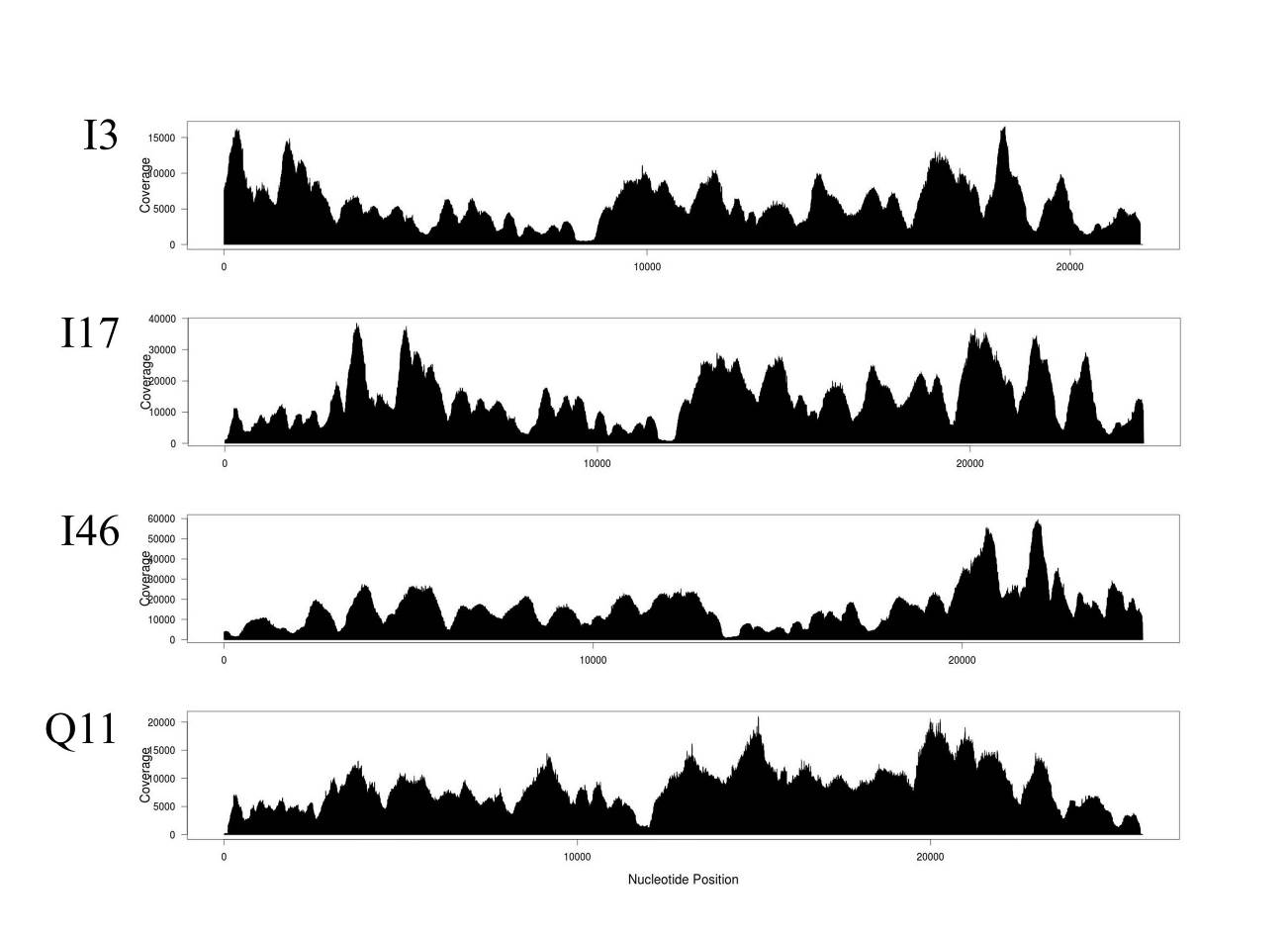


**Figure S6. Coverage distribution of four Q11-like isolates sequenced by *MiSeq* genome sequencer.** X-axis represents the nucleotide position of assembled contig; y-axis represents the coverage on the corresponding position.


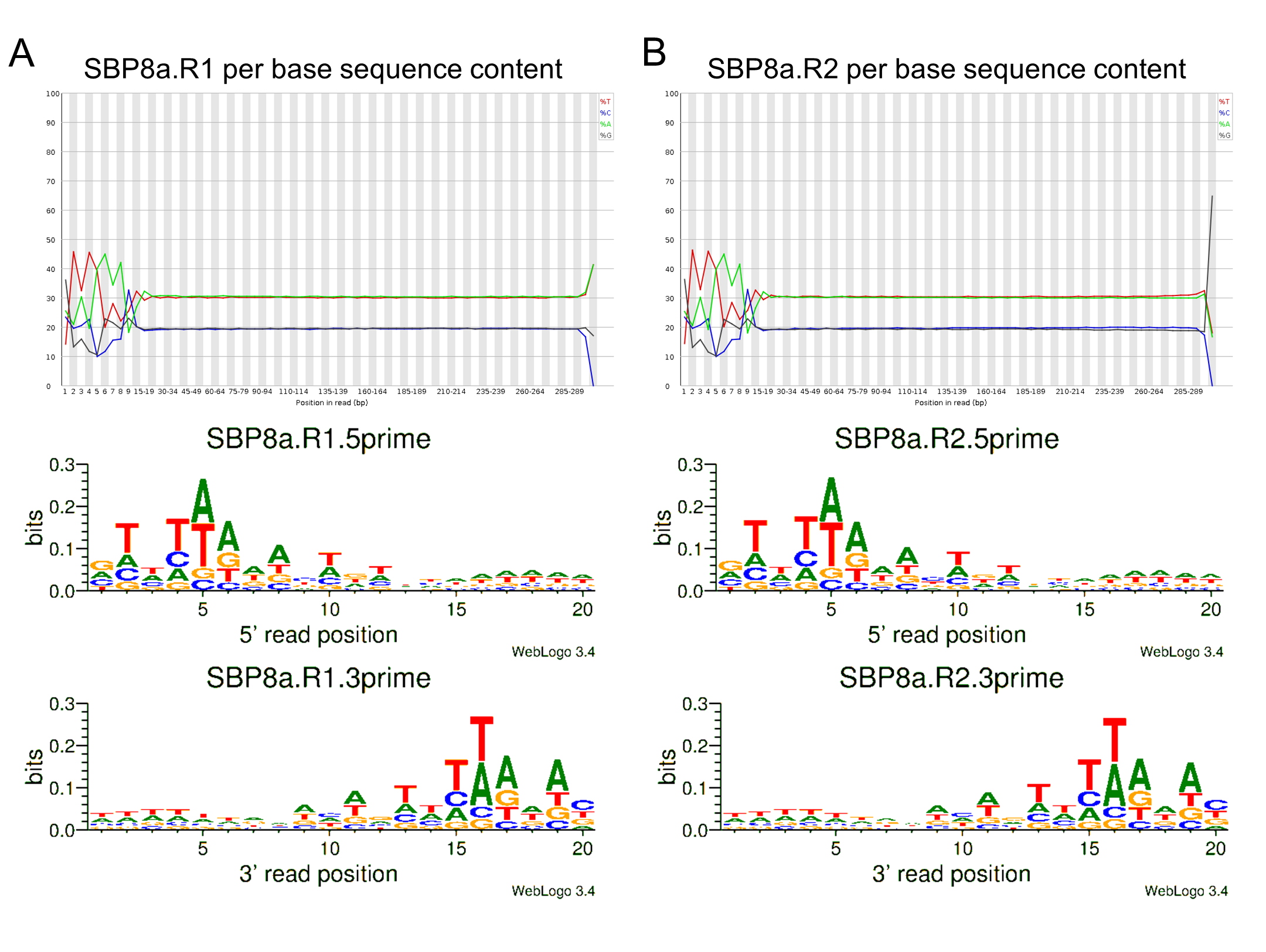


**Figure S7. The sequence content across all bases and sequence logo of first 20bp or last 20 of reads of SBP8a.R1 (A) and SBP8a.R2 (B).** The sequence content was adapted from FastQC. The sequence logo was generated by WebLogo 3.4. The coordinate of 5’ read position represent the first 20 bp of each read in fastq file, while the position 20 of 3’ read position is the last base of each read.


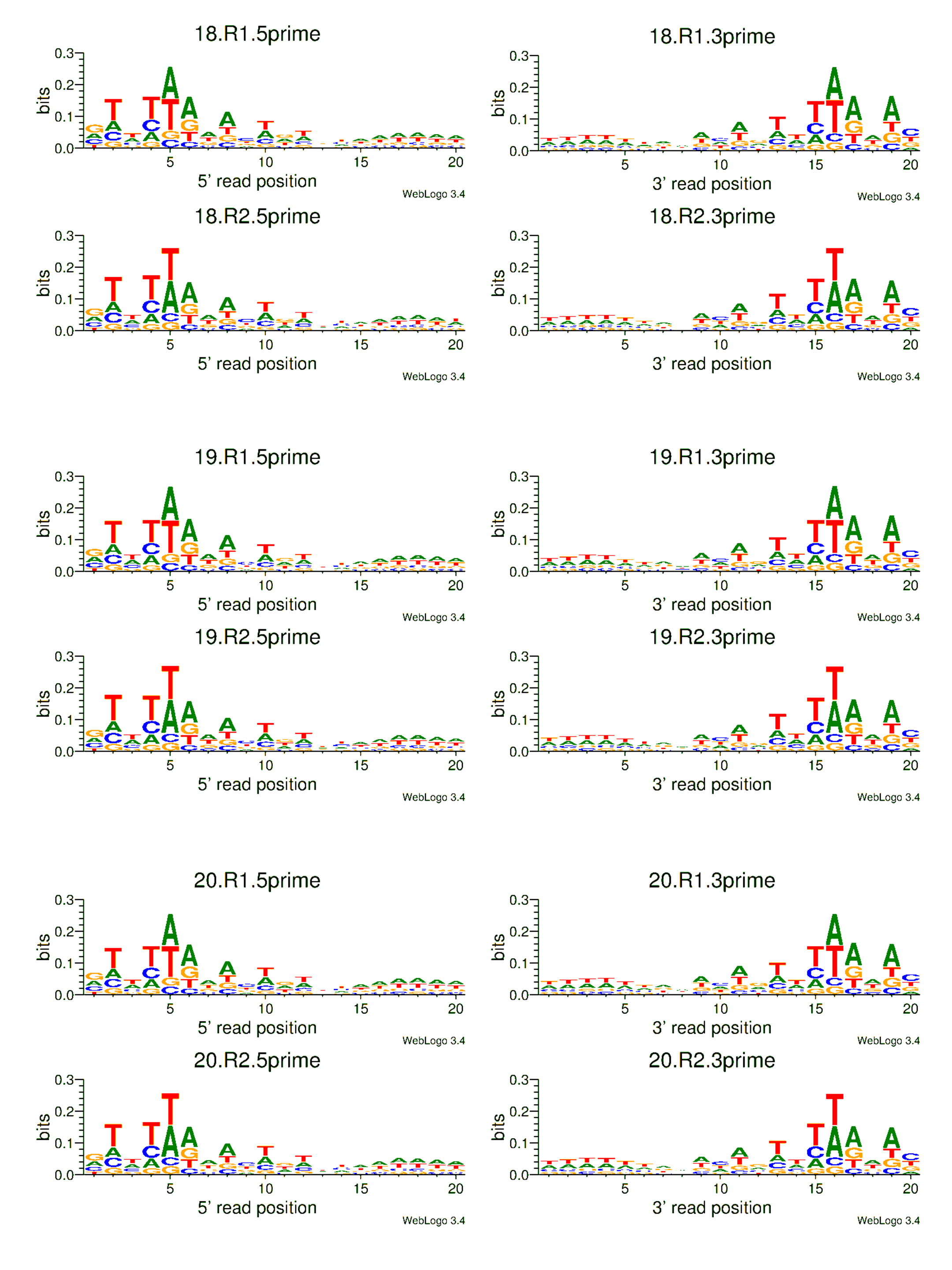


Figure S8 (Continue on next page)


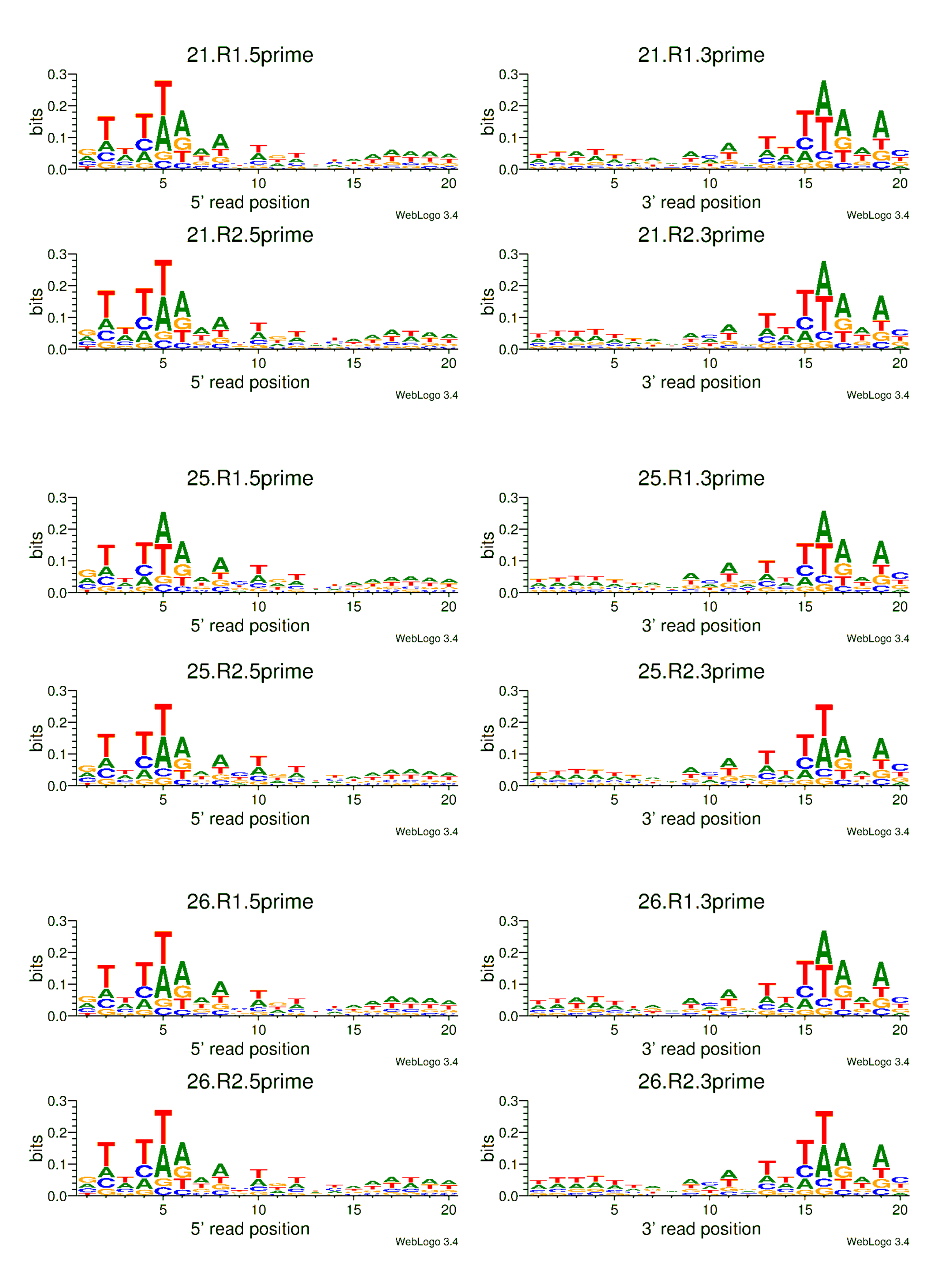


Figure S8 (Continue on next page)


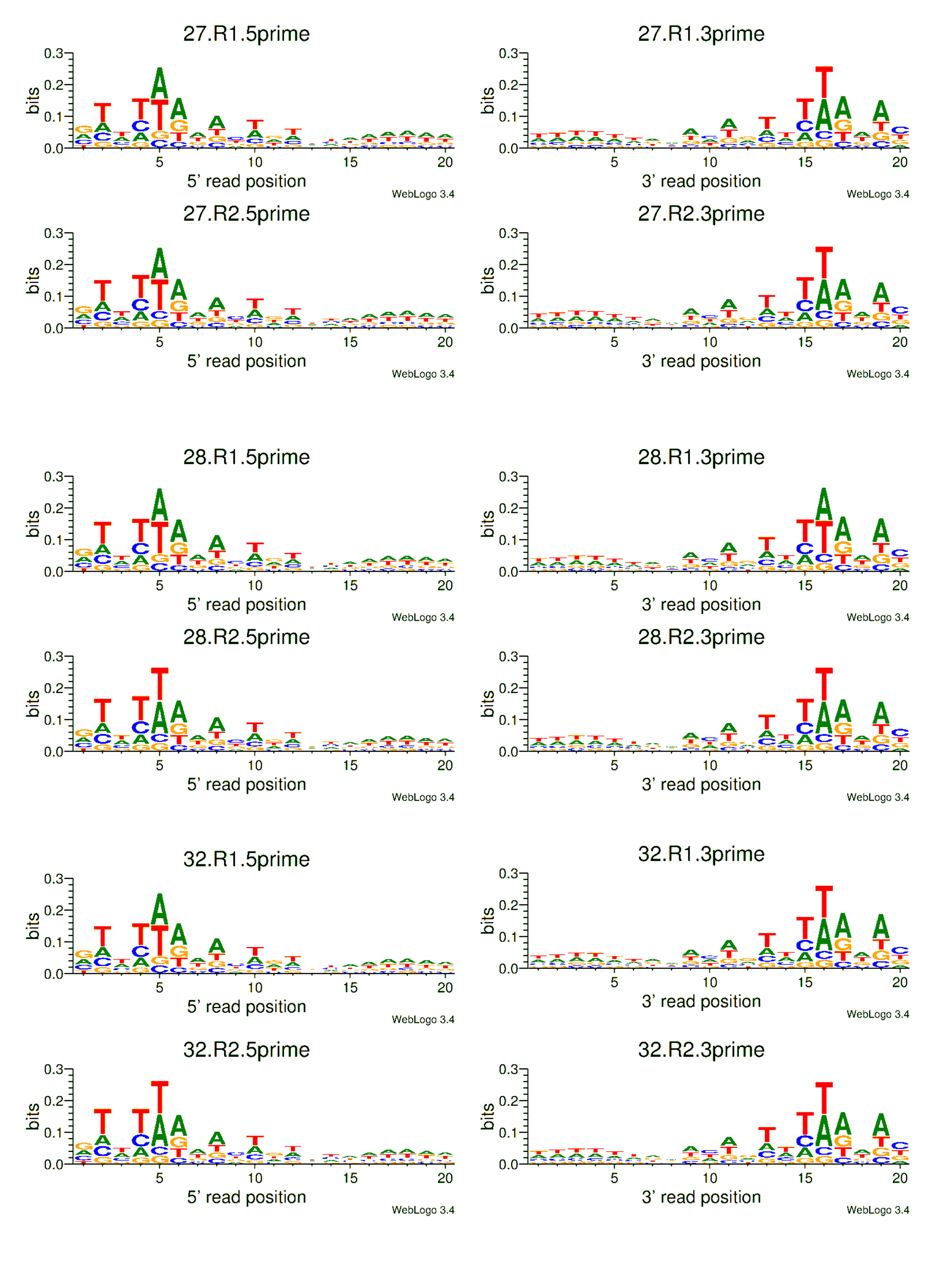


Figure S8 (Continue on next page)


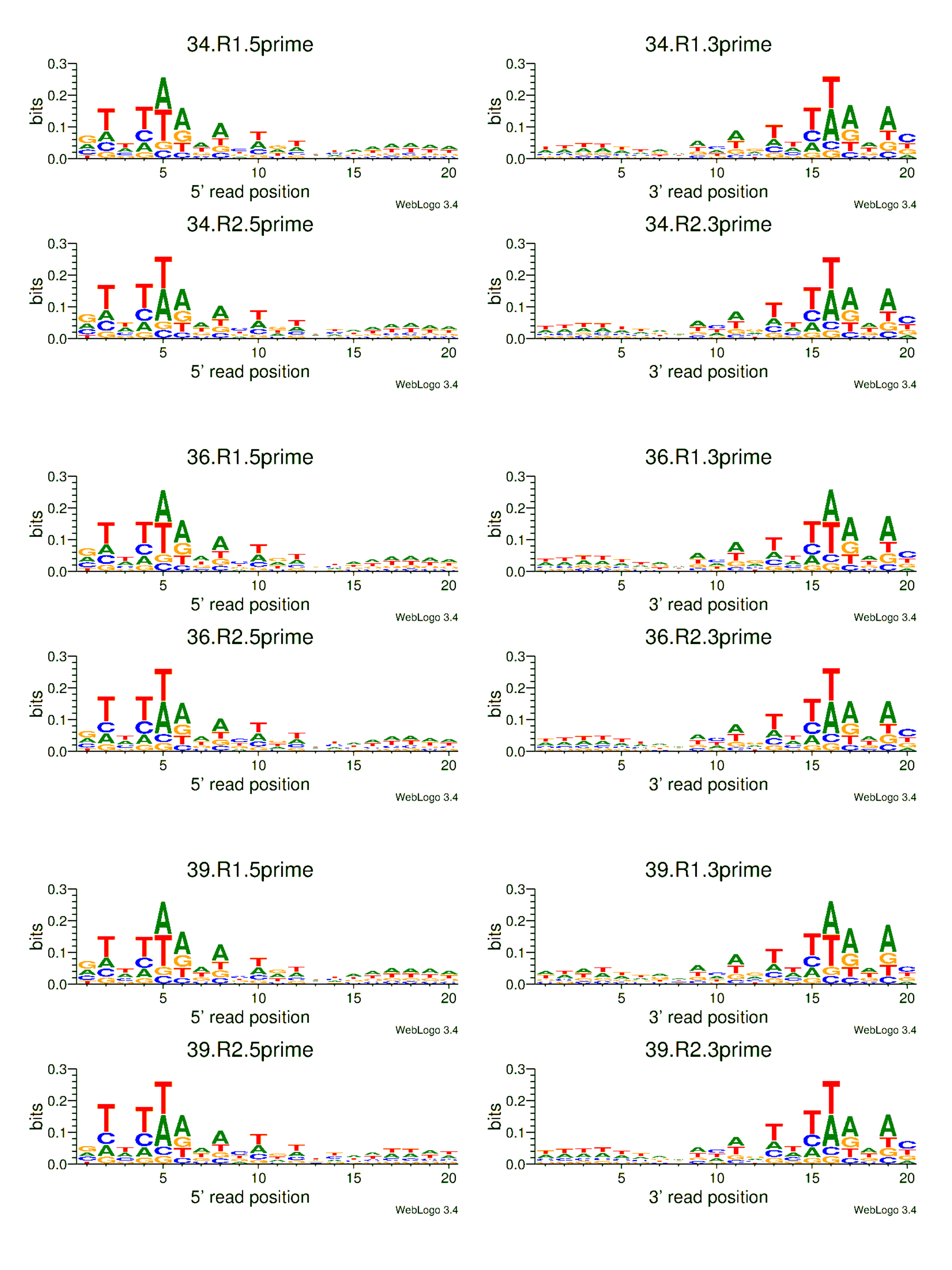


Figure S8 (Continue on next page)


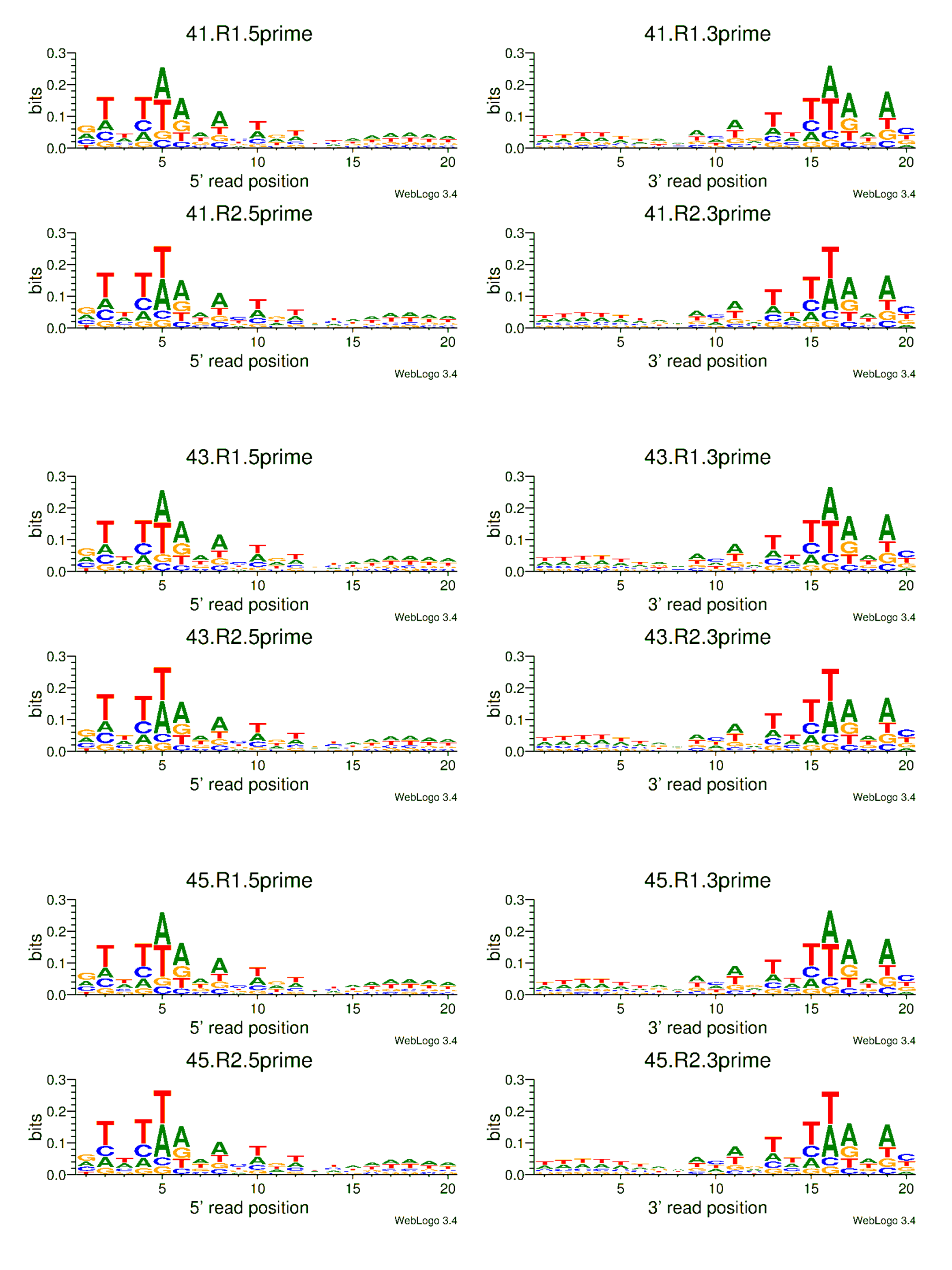


Figure S8 (Continue on next page)


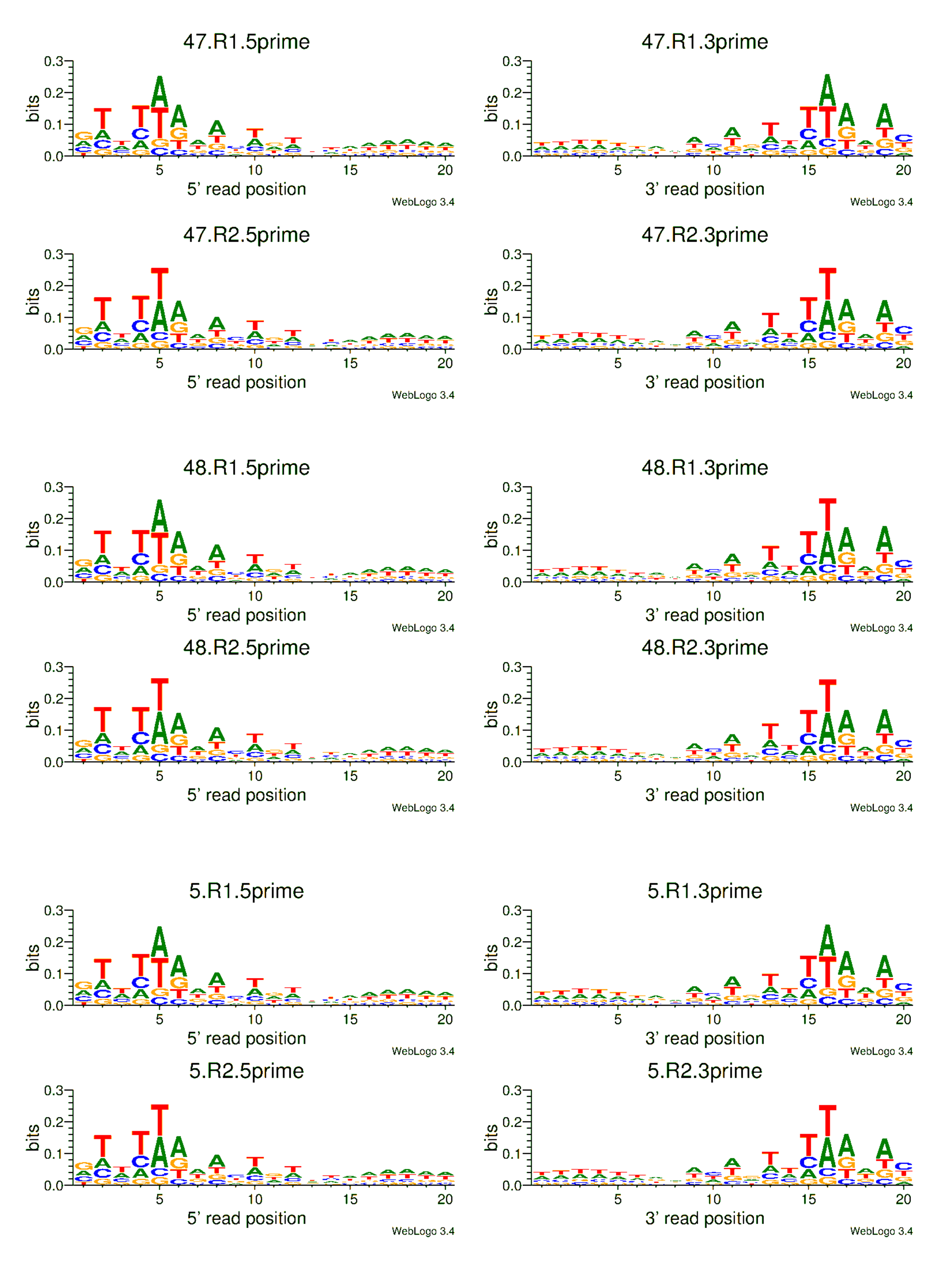


Figure S8 (Continue on next page)


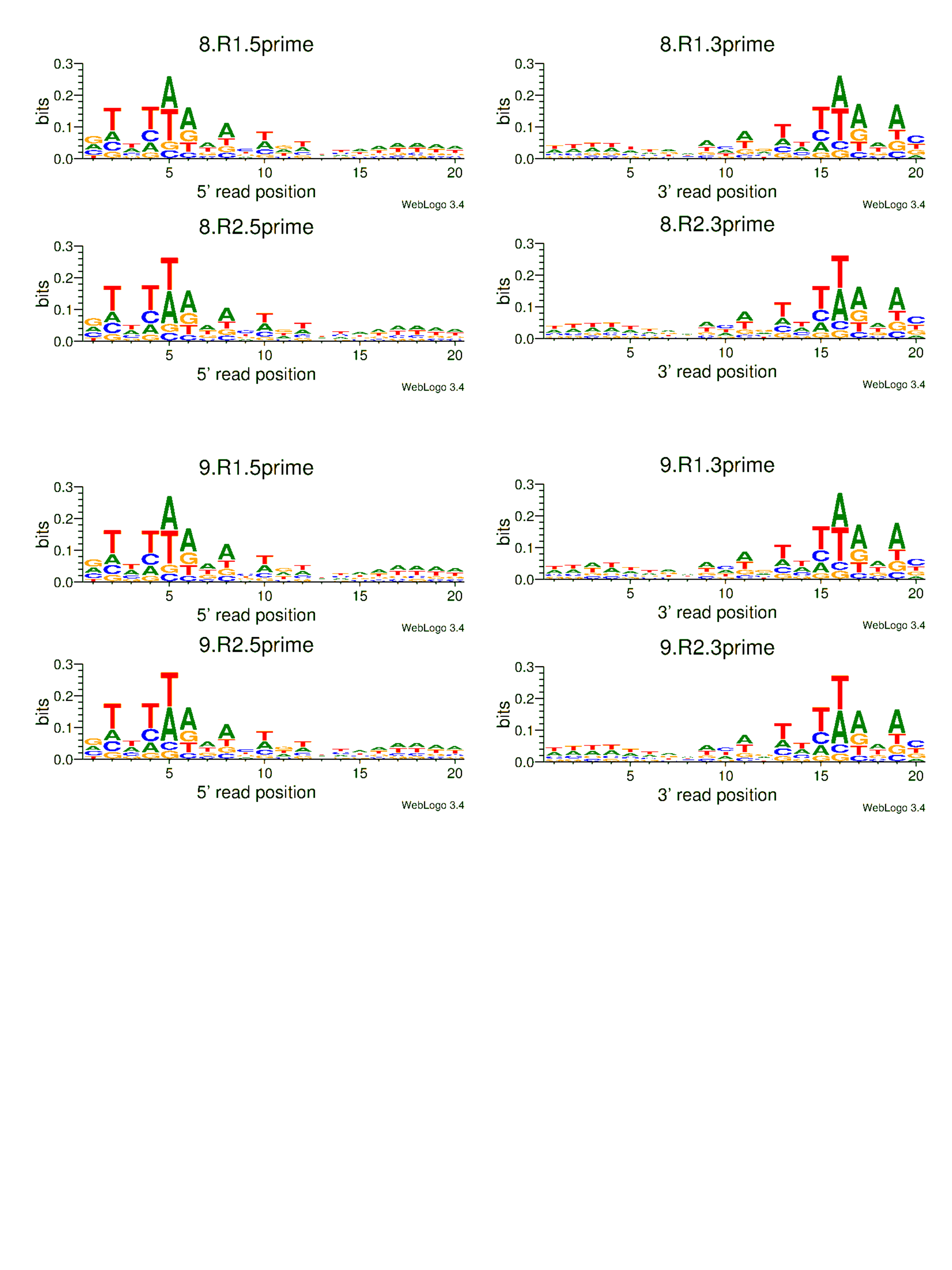


**Figure S8. Sequence logo of I48-like phages sequenced by *MiSeq***. The prefixes of figure labels are the phage isolate numbers in the same order as in Table S3. R1 and R2 represent paired-end read files.


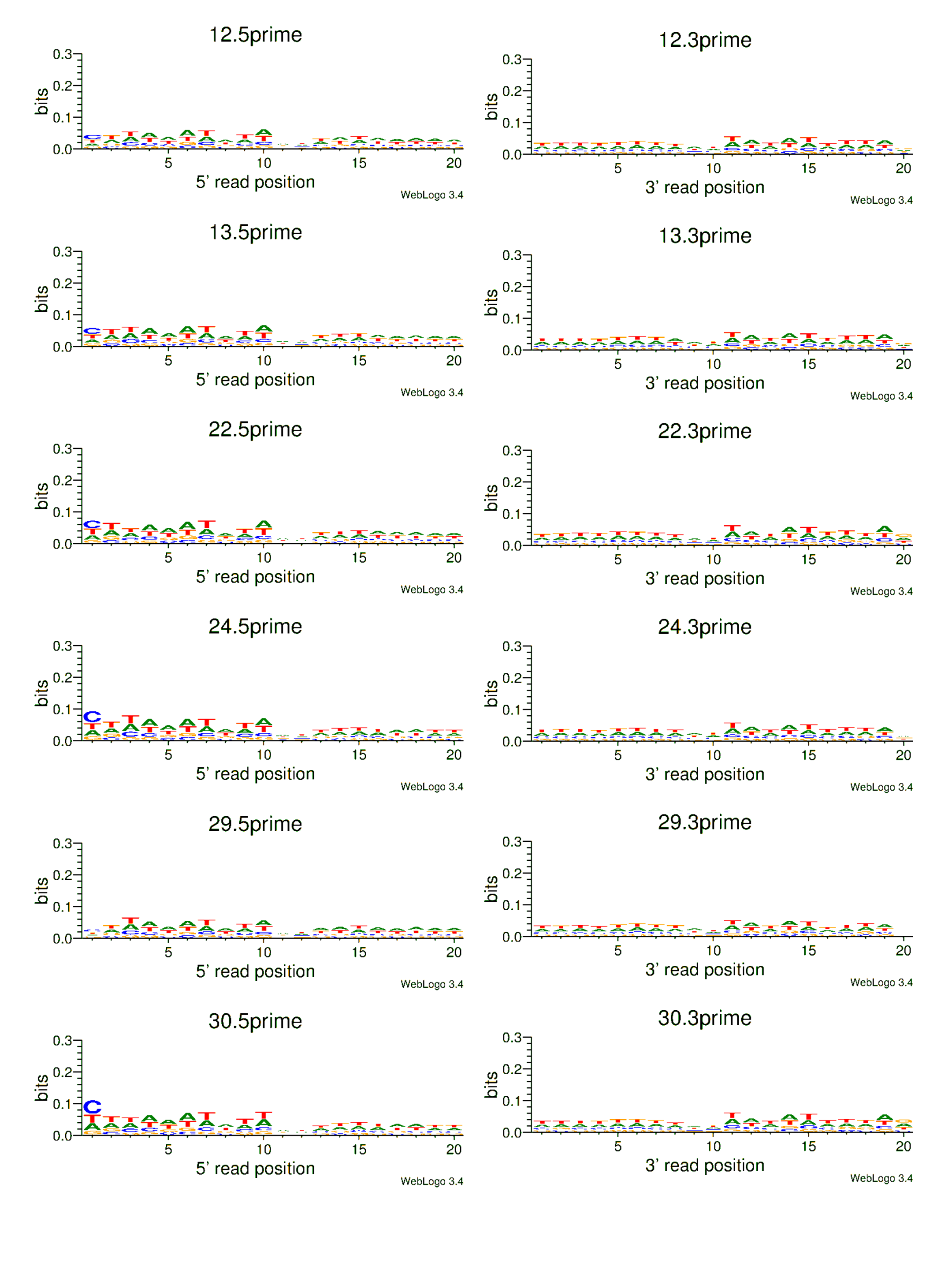


Figure S9 (Continue on next page)


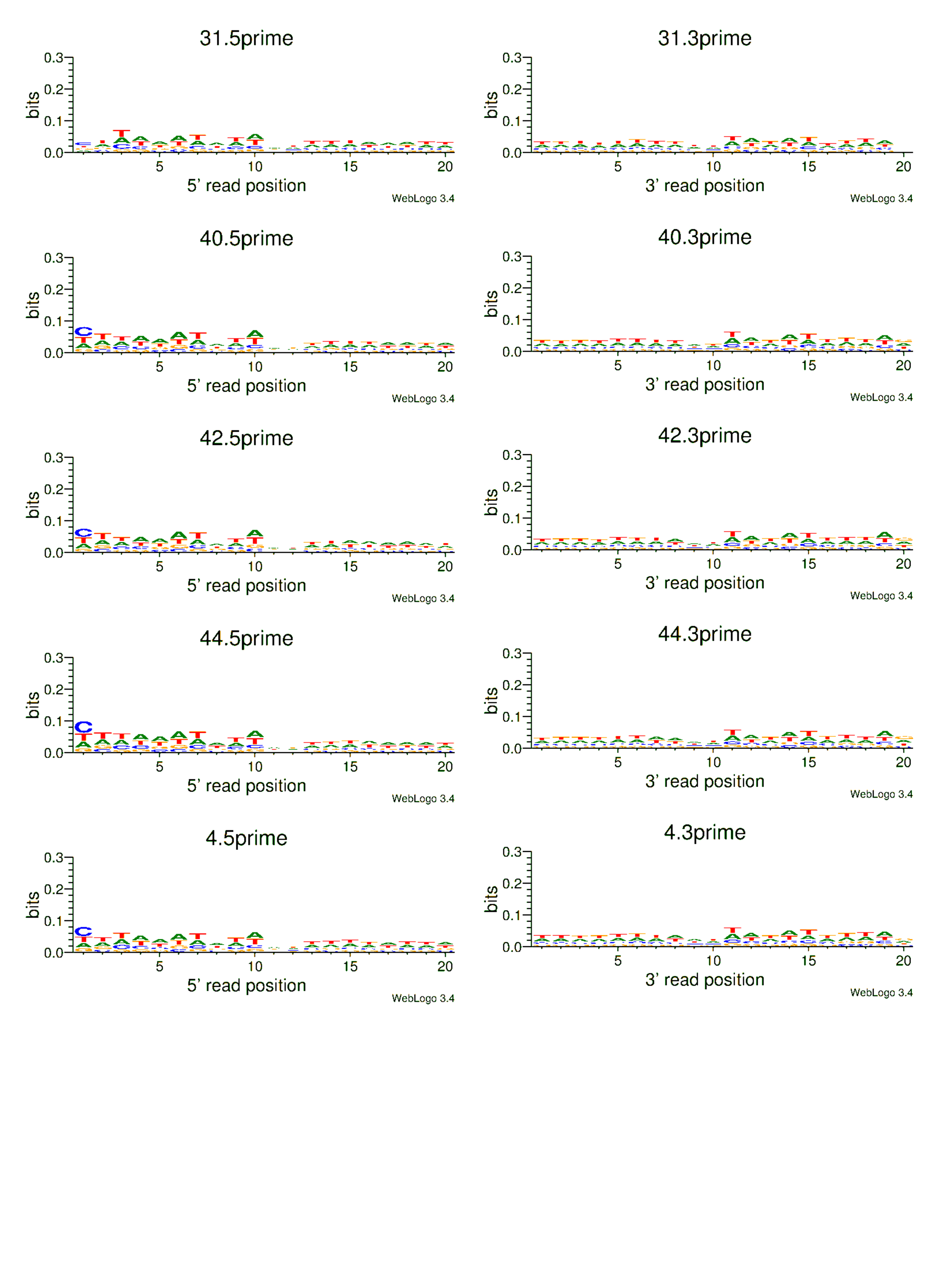


**Figure S9. Sequence logo of I48-like phages sequenced by *PGM*.** The prefixes of figure labels are the phage isolate numbers in the same order as in Table S3. R1 and R2 represent paired-end read files.


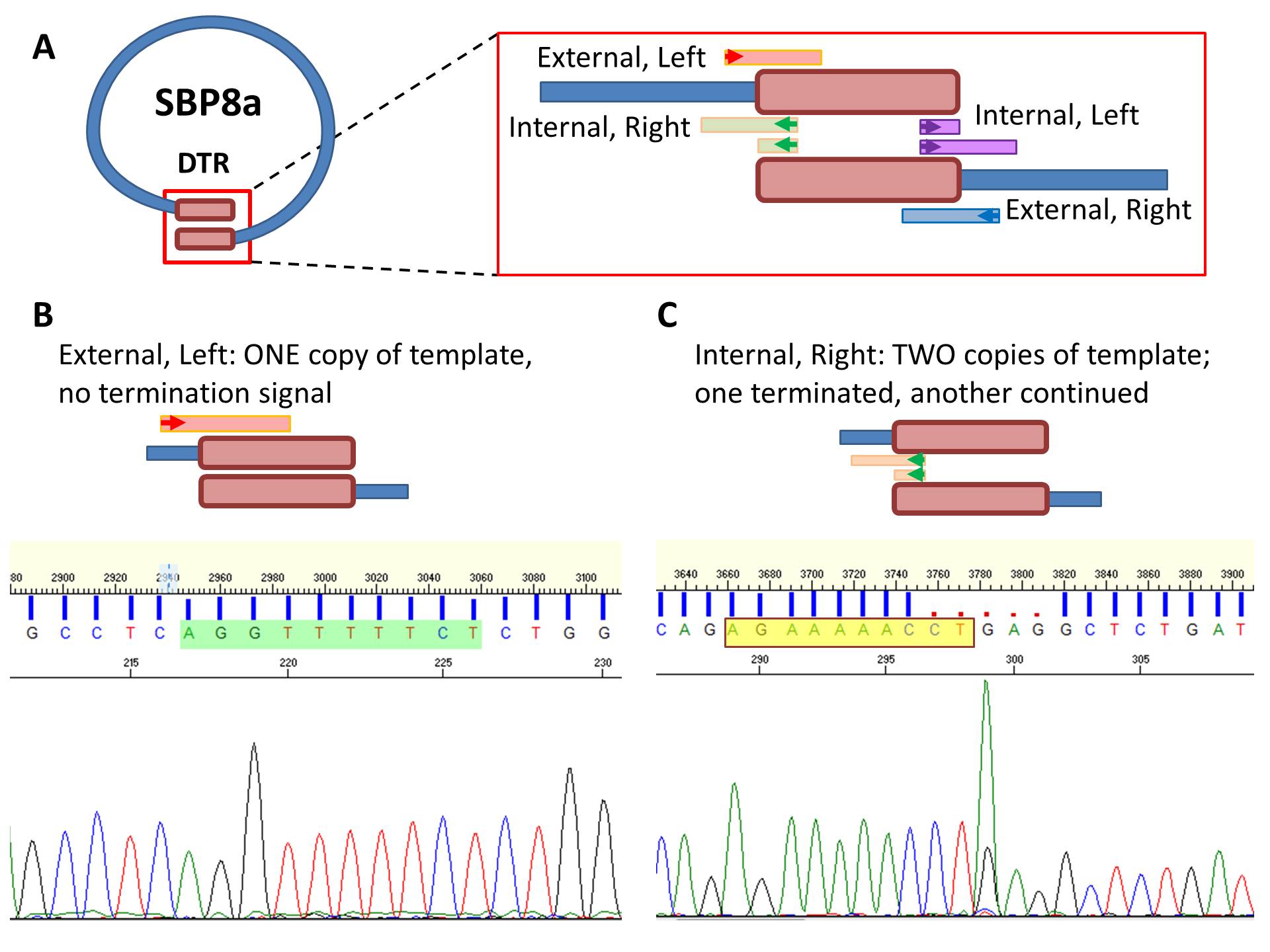
**Figure S10. Example of Sanger sequencing results of SBP8a using primer walking.** SBP8a was predicted as a linear phage with direct terminal repeat (DTR). (A) Illustration of primer design proximate to DTR region. Primers located within DTR region were predicted to observe a sequencing termination occurred followed by a non-templated adenine. (B) Sequencing chromatogram using primer SBP8a-EL (Table S11). (C) Sequencing chromatogram using primer SBP8a-IR. The shadowed sequences 5’-AGGTTTTTCT in (B) and 5’-AGAAAAACCT in (C) was the terminus sequence predicted by NCR and read end frequency developed in this study on position 114,607-114616 of SBP8a contig sequenced by *Roche454*.


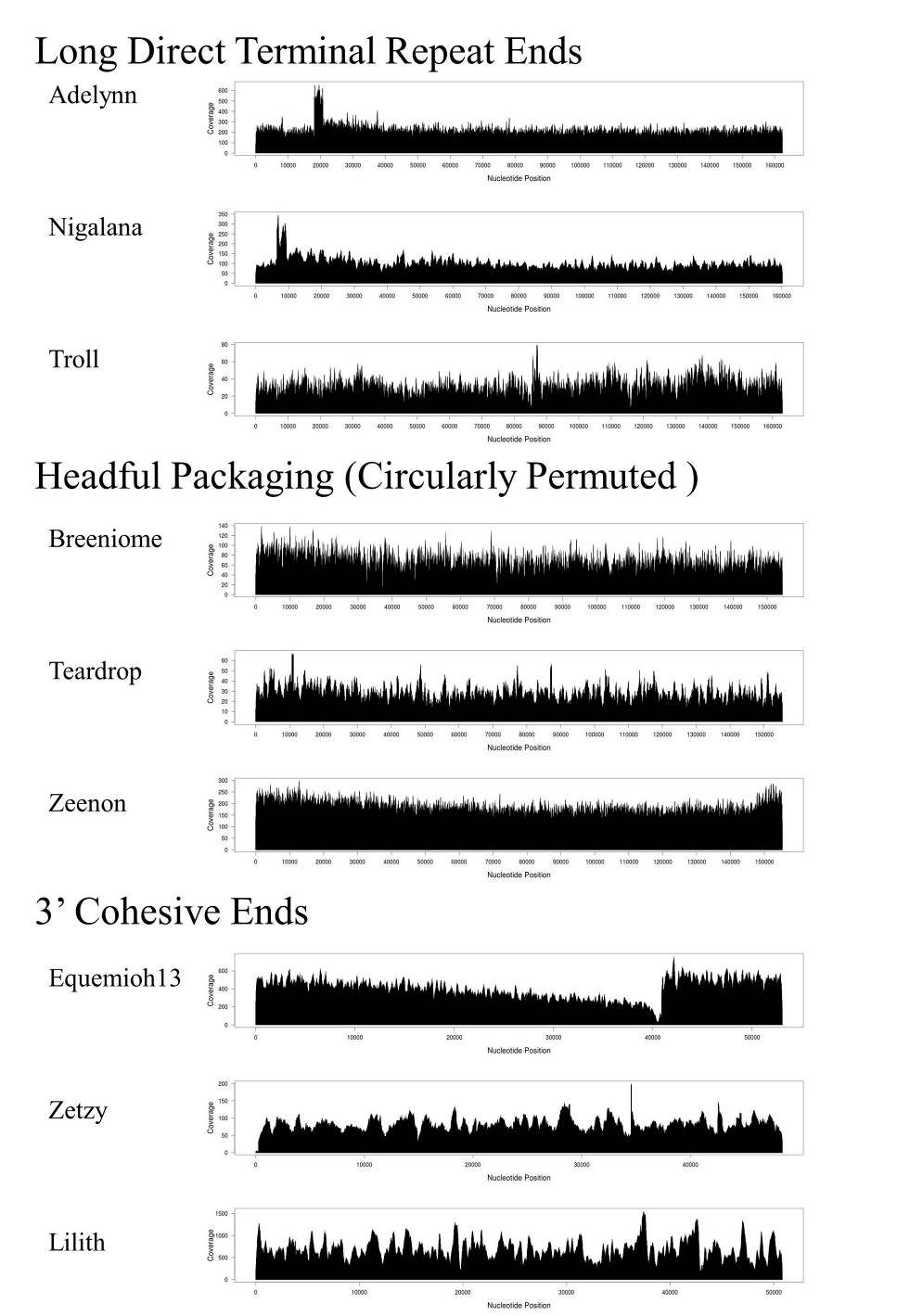


**Figure S11. Coverage distribution of nine previously sequenced phages by *MiSeq, Roche/454 or PGM* genome sequencer.** X-axis represents the nucleotide position of assembled contig; y-axis represents the coverage on the corresponding position.


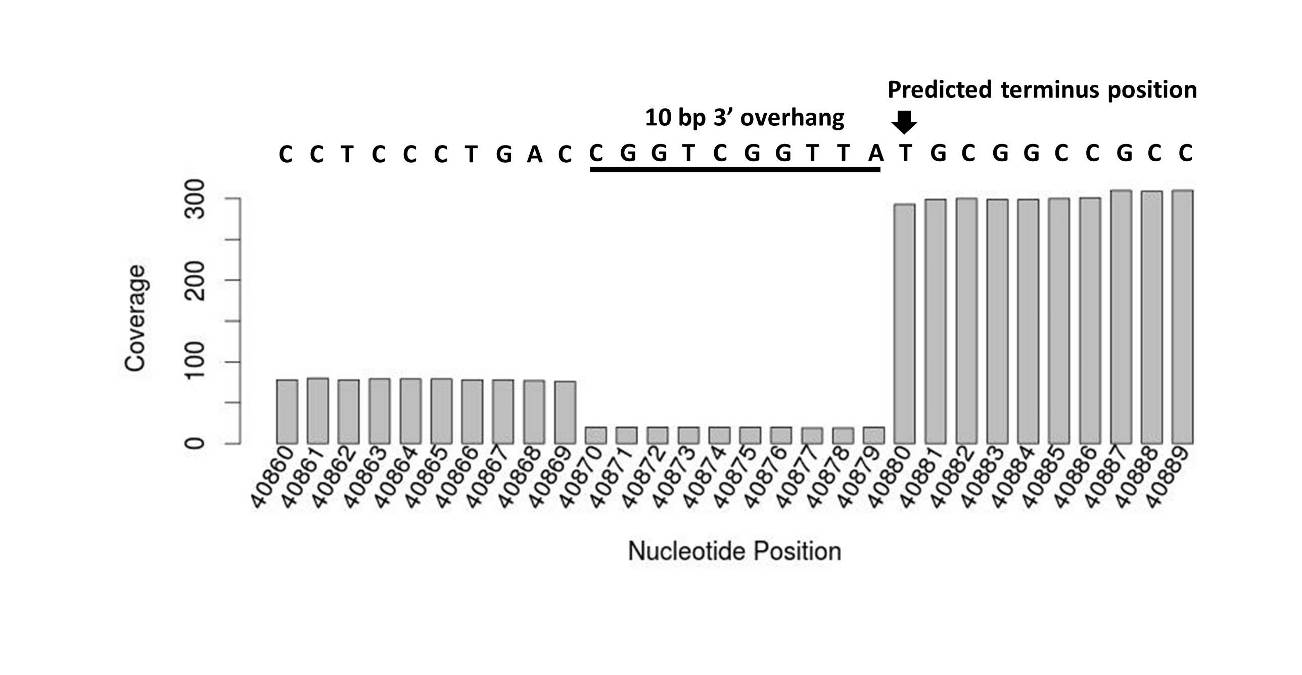


**Figure S12. Coverage distribution of Equemioh13 from contig position 40,860 to 40,889.** The underlined sequences indicate the 10 bp 3’ overhang sequence that was reported on database (http://phagesdb.org/). The significant hit of predicted terminus position on 40,880 is adjoined to the 3’ overhang region.


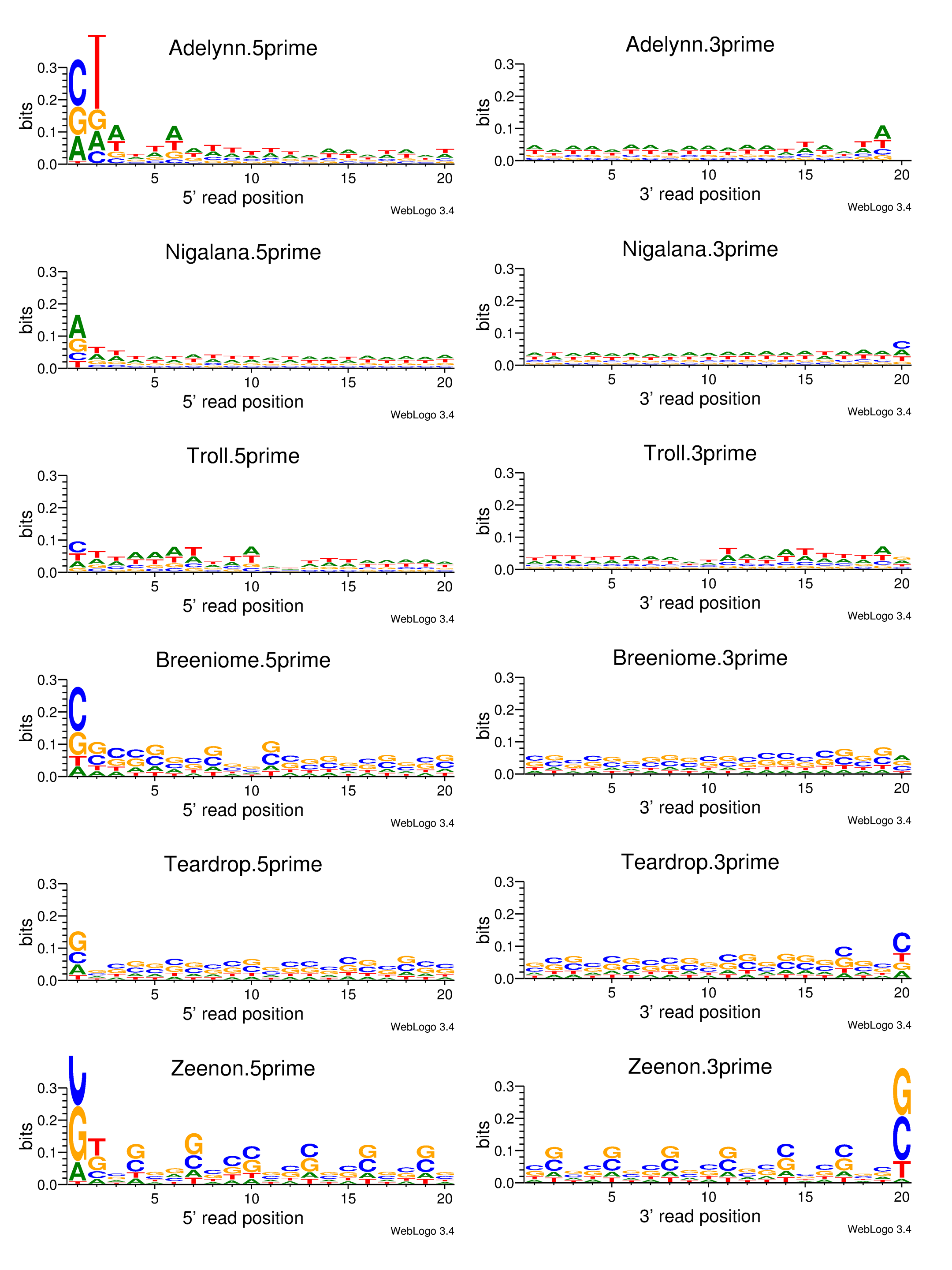


Figure S13 (Continue on next page)


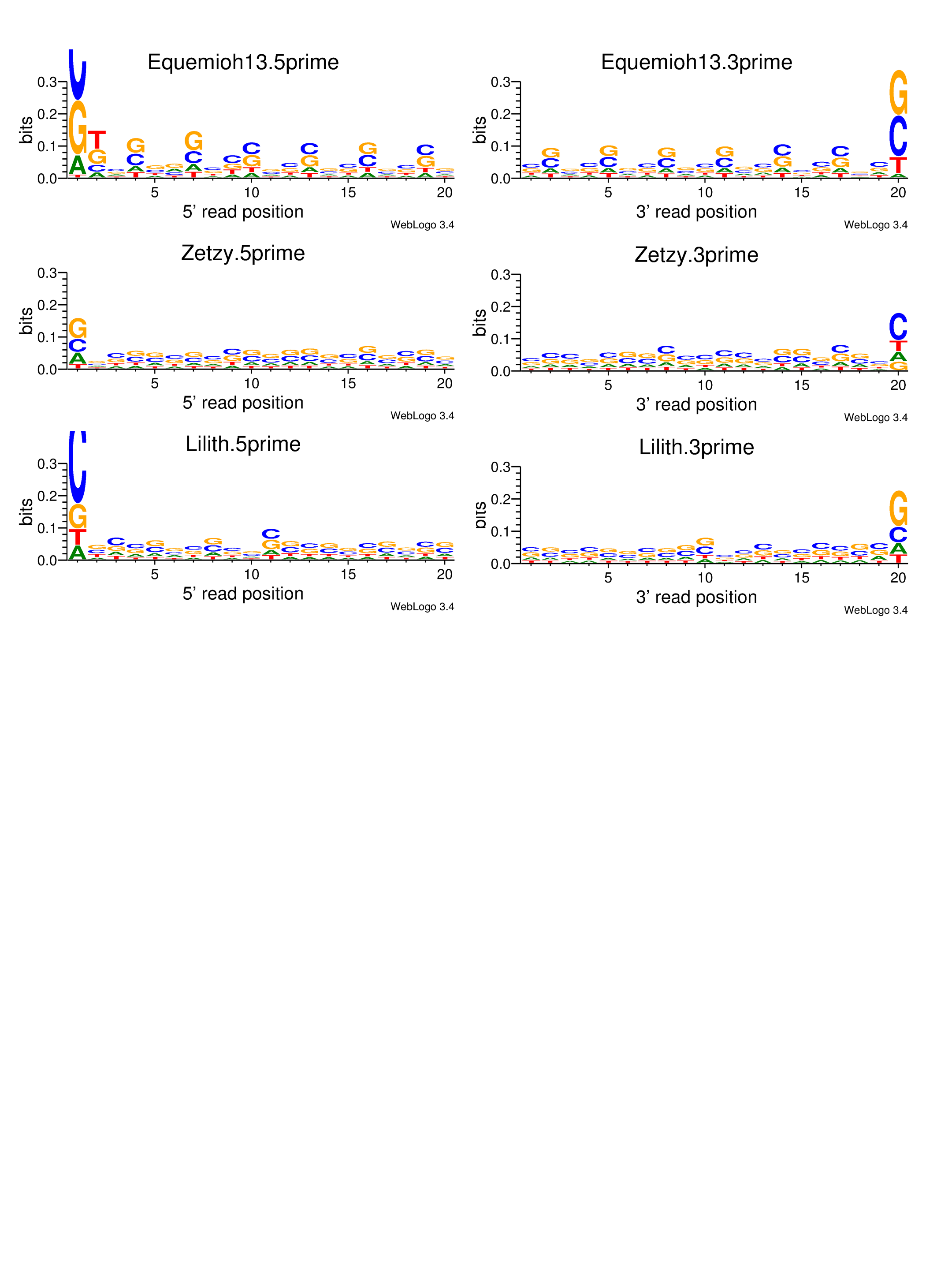


**Figure S13. Sequence logo of nine previously sequenced phages by MiSeq, Roche/454 or PGM genome sequencer.** The prefixes of figure labels are the phage names.

**Table S1. Summary of 23 phage genome assemblies from sequences by *Ion PGM*.**

| Isolates | Read yield | Base yield | Average Length of read | number of contig (>500 bp) | Length of largest contig | Coverage |
| --- | --- | --- | --- | --- | --- | --- |
| I2B | 130478 | 36448132 | 279.34 | 408 | 157773 | 54.27 |
| I12 | 149573 | 45096131 | 301.50 | 1 | 157910 | 276.26 |
| I13 | 186893 | 56984029 | 304.90 | 1 | 157905 | 345.87 |
| I15 | 166588 | 45725098 | 274.48 | 281 | 157854 | 81.34 |
| I22 | 127336 | 37591312 | 295.21 | 1 | 157889 | 235.49 |
| I24 | 192759 | 57331193 | 297.42 | 1 | 157885 | 325.63 |
| I29 | 133229 | 39199737 | 294.23 | 1 | 157878 | 244.76 |
| I30 | 122668 | 34307906 | 279.68 | 1 | 157864 | 214.81 |
| I31 | 151854 | 40604960 | 267.39 | 1 | 157866 | 238.08 |
| I33 | 139978 | 40400219 | 288.62 | 2 | 150224 | 250.21 |
| I35 | 168585 | 49682300 | 294.70 | 2 | 102317 | 310.81 |
| I37 | 161427 | 48105963 | 298.00 | 6 | 156872 | 286.96 |
| I40 | 136464 | 41551515 | 304.49 | 1 | 157892 | 258.58 |
| I42 | 96238 | 30157209 | 313.36 | 1 | 157871 | 188.73 |
| I44 | 171708 | 52082227 | 303.32 | 1 | 157845 | 325.45 |
| I4 | 131860 | 39400251 | 298.80 | 1 | 157919 | 241.91 |
| I6 | 144911 | 42897782 | 296.03 | 517 | 157836 | 55.78 |
| Q10 | 206095 | 61662555 | 299.19 | 1 | 158174 | 383.87 |
| Q11 | 226973 | 67165785 | 295.92 | 2005 | 41047 | 8.09 |
| Q1 | 227943 | 66445270 | 291.50 | 3198 | 131247 | 8.53 |
| Q2 | 202252 | 58905221 | 291.25 | 1 | 158178 | 367.94 |
| Q5 | 180990 | 52939743 | 292.50 | 2 | 126876 | 328.17 |
| Q8 | 72799 | 18992728 | 260.89 | 1 | 158180 | 118.82 |

**Table S2. Summary of 26 phage genome assemblies from sequences by *MiSeq* paired-end sequencing.**

| Isolate | R1 yield | Average Length of R1 | R2 yield | Average Length of R2 | number of contig (>500 bp) | Length of largest contig | Coverage |
| --- | --- | --- | --- | --- | --- | --- | --- |
| I17 | 850507 | 236.85 | 850507 | 237.19 | 1 | 22845 | 6160.02 |
| I18 | 471409 | 245.58 | 471409 | 246.06 | 1 | 157911 | 540.90 |
| I19 | 726027 | 247.80 | 726027 | 248.07 | 1 | 158002 | 830.29 |
| I20 | 512028 | 190.63 | 512028 | 191.11 | 1 | 157756 | 343.74 |
| I21 | 953536 | 217.41 | 953536 | 217.48 | 1 | 158063 | 744.83 |
| I25 | 972340 | 208.21 | 972340 | 208.50 | 1 | 157912 | 841.63 |
| I26 | 1296142 | 208.05 | 1296142 | 208.64 | 1 | 157714 | 1092.74 |
| I27 | 1348561 | 202.70 | 1348561 | 204.31 | 1 | 157886 | 1029.18 |
| I28 | 946695 | 229.80 | 946695 | 230.48 | 1 | 157772 | 989.90 |
| I3 | 330818 | 218.72 | 330818 | 217.84 | 1 | 21717 | 2077.66 |
| I32 | 750439 | 271.95 | 750439 | 271.74 | 1 | 157823 | 947.10 |
| I34 | 2339966 | 220.97 | 2339966 | 221.96 | 1 | 157851 | 2195.75 |
| I36 | 862625 | 256.57 | 862625 | 256.43 | 1 | 157701 | 1096.35 |
| I39 | 548444 | 255.90 | 548444 | 255.28 | 1 | 157715 | 721.49 |
| I41 | 802973 | 233.30 | 802973 | 233.39 | 1 | 157855 | 860.46 |
| I43 | 1050199 | 267.06 | 1050199 | 266.77 | 1 | 157857 | 1402.70 |
| I45 | 1686621 | 252.02 | 1686621 | 253.10 | 1 | 157713 | 1908.72 |
| I46 | 1693118 | 237.74 | 1693118 | 237.83 | 1 | 24896 | 12345.86 |
| I47 | 1435984 | 219.28 | 1435984 | 219.92 | 1 | 157787 | 1380.61 |
| I48 | 1487589 | 249.58 | 1487589 | 249.85 | 1 | 158046 | 1748.00 |
| I50 | 962336 | 229.31 | 962336 | 229.52 | 2 | 158044 | 789.02 |
| I5 | 971776 | 219.53 | 971776 | 220.07 | 1 | 157985 | 905.78 |
| I8 | 977737 | 221.90 | 977737 | 222.40 | 1 | 158042 | 963.86 |
| I9 | 455199 | 227.39 | 455199 | 228.58 | 1 | 157856 | 356.15 |
| Q11 | 1205764 | 189.69 | 1205764 | 189.83 | 1 | 26005 | 5342.89 |
| SPB8a | 2331088 | 173.27 | 2331088 | 173.78 | 1 | 158951 | 1353.72 |

**Table S3. Genome terminus prediction of 31 I48-like isolates using NGS data.**

|  |  |  |  |  |  | NCR |  |  |  |  |  |  |  | Read edge frequency | |  |  |  |  |  |
| --- | --- | --- | --- | --- | --- | --- | --- | --- | --- | --- | --- | --- | --- | --- | --- | --- | --- | --- | --- | --- |
| Sequencer | Isolate | Genome Size | Contig form | Coverage | 1.8 FC | L coverage | L start | L end | R coverage | R start | R end | Coverage ratio |  | 5' edge position | Frequency | flanking sequence | 3' edge position | Frequency | flanking sequence | Size of DTR |
| PGM | I12 | 157910 | Circular | 275.18 | 495.32 | 285.73 | 68887 | 68986 | 655.91 | **68987** | 69086 | **2.296** |  | 65061 | 116 |  | **71748** | **216** | AGAAAAACCT-3' | 2750 |
|  |  |  |  |  |  | 528.02 | 71649 | **71748** | 158.98 | 71749 | 71848 | **0.301** |  | **68998** | **114** | 5'-AAACCGTATG | 156889 | 64 |  |  |
|  |  |  |  |  |  |  |  |  |  |  |  |  |  | 64312 | 53 |  | 71747 | 50 |  |  |
| PGM | I13 | 157905 | Circular | 345.92 | 622.66 | 400.11 | 111500 | 111599 | 814.75 | **111600** | 111699 | **2.036** |  | **111610** | **110** | 5'-AAACCGTATG | **114359** | **274** | AGAAAAACCT-3' | 2749 |
|  |  |  |  |  |  | 840.61 | 114260 | **114359** | 352.68 | 114360 | 114459 | **0.42** |  | 107673 | 71 |  | 114358 | 93 |  |  |
|  |  |  |  |  |  |  |  |  |  |  |  |  |  | 111537 | 63 |  | 41587 | 73 |  |  |
| PGM | I22 | 157889 | Circular | 229.43 | 412.98 | 245.47 | 101642 | 101741 | 548.76 | 101742 | 101841 | 2.236 |  | **106421** | **221** | 5'-AAACCGTATG | **109166** | **864** | CCAGAGAAAA-3' | 2745 |
|  |  |  |  |  |  | 545.48 | 106313 | 106412 | 1091.87 | **106413** | 106512 | **2.002** |  | 102485 | 162 |  | 109169 | 47 | AGAAAAACCT-3' |  |
|  |  |  |  |  |  | 1199.59 | 109063 | **109162** | 96.25 | 109163 | 109262 | **0.08** |  | 106222 | 97 |  | 83473 | 37 |  |  |
| PGM | I24 | 157885 | Circular | 343.21 | 617.78 | 370.93 | 3962 | 4061 | 810.86 | **4062** | 4161 | **2.186** |  | **4072** | **114** | 5'-AAACCGTATG | **6821** | **193** | AGAAAAACCT-3' | 2749 |
|  |  |  |  |  |  | 687.41 | 6722 | **6821** | 354.31 | 6822 | 6921 | **0.515** |  | 137 | 89 |  | 91943 | 55 |  |  |
|  |  |  |  |  |  |  |  |  |  |  |  |  |  | 3999 | 58 |  | 6820 | 49 |  |  |
| PGM | I29 | 157878 | Circular | 240.61 | 433.10 | 270.82 | 130387 | 130486 | 677.08 | **130487** | 130586 | **2.5** |  | **130501** | **114** | 5'-AAACCGTATG | **133251** | **380** | AGAAAAACCT-3' | 2750 |
|  |  |  |  |  |  | 795.58 | 133152 | **133251** | 244.41 | 133252 | 133351 | **0.307** |  | 130428 | 102 |  | 60471 | 63 |  |  |
|  |  |  |  |  |  |  |  |  |  |  |  |  |  | 126563 | 78 |  | 133250 | 49 |  |  |
| PGM | I30 | 157864 | Circular | 211.04 | 379.87 | 209.41 | 173 | 272 | 397.13 | 273 | 372 | 1.896 |  | **883** | **197** | 5'-AAACCGTATG | **3632** | **852** | AGAAAAACCT-3' | 2749 |
|  |  |  |  |  |  | 524.39 | 773 | 872 | 1009.19 | **873** | 972 | **1.925** |  | 154809 | 143 |  | 3631 | 182 |  |  |
|  |  |  |  |  |  | 1375.04 | 3533 | **3632** | 46.21 | 3633 | 3732 | **0.034** |  | 683 | 88 |  | 3630 | 50 |  |  |
|  |  |  |  |  |  | 233.8 | 153966 | 154065 | 532.79 | 154066 | 154165 | 2.279 |  |  |  |  |  |  |  |  |
| PGM | I31 | 157866 | Circular | 246.09 | 442.97 | 243.08 | 78611 | 78710 | 532.76 | **78711** | 78810 | **2.192** |  | **78720** | **120** | 5'-AAACCGTATG | **81469** | **161** | AGAAAAACCT-3' | 2749 |
|  |  |  |  |  |  | 483.95 | 81370 | **81469** | 205.9 | 81470 | 81569 | **0.425** |  | 74782 | 81 |  | 8689 | 41 |  |  |
|  |  |  |  |  |  |  |  |  |  |  |  |  |  | 78521 | 48 |  | 88762 | 32 |  |  |
| PGM | I40 | 157892 | Circular | 252.48 | 454.46 | 202.37 | 37157 | 37256 | 465.31 | 37257 | 37356 | 2.299 |  | **41935** | **211** | 5'-AAACCGTATG | **44680** | **479** | AGAAAAACCT-3' | 2745 |
|  |  |  |  |  |  | 485.58 | 41825 | 41924 | 1027.15 | **41925** | 42024 | **2.115** |  | 38000 | 119 |  | 129813 | 62 |  |  |
|  |  |  |  |  |  | 725.15 | 44577 | **44676** | 78.36 | 44677 | 44776 | **0.108** |  | 41933 | 81 |  | 151399 | 36 |  |  |
| PGM | I42 | 157871 | Circular | 184.19 | 331.54 | 177.48 | 11349 | 11448 | 373.87 | **11449** | 11548 | **2.107** |  | **11459** | **57** | 5'-AAACCGTATG | **14207** | **116** | AGAAAAACCT-3' | 2748 |
|  |  |  |  |  |  | 304.98 | 14108 | **14207** | 122.52 | 14208 | 14307 | **0.402** |  | 7522 | 27 |  | 99298 | 39 |  |  |
|  |  |  |  |  |  |  |  |  |  |  |  |  |  | 54441 | 26 |  | 120889 | 30 |  |  |
| PGM | I44 | 157845 | Circular | 317.89 | 572.21 | 384.71 | 63829 | 63928 | 853.12 | **63929** | 64028 | **2.218** |  | **63943** | **142** | 5'-AAACCGTATG | **66692** | **317** | AGAAAAACCT-3' | 2749 |
|  |  |  |  |  |  | 772.23 | 66593 | **66692** | 258.02 | 66693 | 66792 | **0.334** |  | 60007 | 107 |  | 66691 | 80 |  |  |
|  |  |  |  |  |  |  |  |  |  |  |  |  |  | 63870 | 74 |  | 151773 | 71 |  |  |
| PGM | I4 | 157919 | Circular | 240.17 | 432.30 | 224.56 | 34940 | 35039 | 524.3 | **35040** | 35139 | **2.335** |  | **35050** | **83** | 5'-AAACCGTATG | **37799** | **139** | AGAAAAACCT-3' | 2749 |
|  |  |  |  |  |  | 478.61 | 37700 | **37799** | 211.47 | 37800 | 37899 | **0.442** |  | 31112 | 65 |  | 122939 | 60 |  |  |
|  |  |  |  |  |  |  |  |  |  |  |  |  |  | 35048 | 37 |  | 37798 | 52 |  |  |
| MiSeq | I18 | 157911 | Circular | 1277.56 | 2299.61 | 2033.25 | 13288 | 13387 | 4109.29 | 13388 | 13487 | 2.021 |  | 13438 | 631 | 5'-GTATAAAGGA | 14079 | 574 | ACTTCATGAT-3' |  |
|  |  |  |  |  |  | 2978.71 | 14576 | 14675 | 1376.35 | 14676 | 14775 | 0.462 |  | 15881 | 611 |  | 13727 | 560 |  |  |
|  |  |  |  |  |  | 3560.73 | 16019 | 16118 | 1432.9 | 16119 | 16218 | 0.402 |  | 13822 | 604 |  | 14230 | 484 |  |  |
| MiSeq | I19 | 157866 | Circular | 2003.08 | 3605.54 | 9713.33 | 74063 | 74162 | 4456.9 | 74163 | 74262 | 0.459 |  | 73308 | 1915 | ATATAATAGT | 73565 | 1787 | ACTTCATGAT |  |
|  |  |  |  |  |  | 9779.37 | 75512 | 75611 | 1666.96 | 75612 | 75711 | 0.17 |  | 72924 | 1762 |  | 73213 | 1533 |  |  |
|  |  |  |  |  |  |  |  |  |  |  |  |  |  | 75367 | 1761 |  | 75375 | 1427 |  |  |
| MiSeq | I20 | 157756 | Circular | 1172.60 | 2110.68 | 1307.44 | 25751 | 25850 | 2363.28 | 25851 | 25950 | 1.808 |  | 31466 | 1508 | GTTCTAGGGA | 31474 | 1433 | TGTTCTAGGG |  |
|  |  |  |  |  |  | 4048.63 | 30160 | 30259 | 1876.14 | 30260 | 30359 | 0.463 |  | 31358 | 855 |  | 29664 | 845 |  |  |
|  |  |  |  |  |  | 4515.82 | 31192 | 31291 | 8291.69 | 31292 | 31391 | 1.836 |  | 31264 | 842 |  | 31423 | 735 |  |  |
|  |  |  |  |  |  | 4756.88 | 31616 | 31715 | 782.34 | 31716 | 31815 | 0.164 |  |  |  |  |  |  |  |  |
| MiSeq | I21 | 157912 | Circular | 1330.90 | 2395.62 | 26947.26 | 19313 | 19412 | 11786.11 | 19413 | 19512 | 0.437 |  | 20617 | 10789 | GTTCTAGGGA | 20625 | 9804 | TGTTCTAGGG |  |
|  |  |  |  |  |  | 21668.23 | 20768 | 20867 | 421.24 | 20868 | 20967 | 0.019 |  | 20566 | 7824 |  | 18815 | 8507 |  |  |
|  |  |  |  |  |  |  |  |  |  |  |  |  |  | 18558 | 7736 |  | 20574 | 7195 |  |  |
| MiSeq | I25 | 157912 | Circular | 1291.70 | 2325.06 | 1703.38 | 29009 | 29108 | 3176.44 | 29109 | 29208 | 1.865 |  | 31602 | 1563 | GTTCTAGGGA | 31610 | 1312 | TGTTCTAGGG |  |
|  |  |  |  |  |  | 2867.16 | 30298 | 30397 | 1513.71 | 30398 | 30497 | 0.528 |  | 29159 | 894 |  | 29800 | 1098 |  |  |
|  |  |  |  |  |  | 3497.77 | 31741 | 31840 | 1028.73 | 31841 | 31940 | 0.294 |  | 31428 | 824 |  | 29951 | 925 |  |  |
| MiSeq | I26 | 157714 | Circular | 3154.20 | 5677.55 | 51563.21 | 30210 | 30309 | 25446.17 | 30310 | 30409 | 0.493 |  | 31515 | 12188 | GTTCTAGGGA | 31523 | 12023 | TGTTCTAGGG |  |
|  |  |  |  |  |  | 36293.41 | 31668 | 31767 | 1136.86 | 31768 | 31867 | 0.031 |  | 31351 | 8486 |  | 29713 | 8329 |  |  |
|  |  |  |  |  |  |  |  |  |  |  |  |  |  | 31313 | 8466 |  | 31472 | 7975 |  |  |
| MiSeq | I27 | 157886 | Circular | 3200.16 | 5760.29 | 9270.49 | 30299 | 30398 | 4683.81 | 30399 | 30498 | 0.505 |  | 31592 | 2432 | GTTCTAGGGA | 31600 | 2531 | TGTTCTAGGG |  |
|  |  |  |  |  |  | 9887.3 | 31731 | 31830 | 2184.55 | 31831 | 31930 | 0.221 |  | 31428 | 1689 |  | 29790 | 1607 |  |  |
|  |  |  |  |  |  |  |  |  |  |  |  |  |  | 31390 | 1524 |  | 31549 | 1450 |  |  |
| MiSeq | I28 | 157772 | Circular | 1388.84 | 2499.92 | 3183.38 | 30197 | 30296 | 1468.49 | 30297 | 30396 | 0.461 |  | 31502 | 1484 | GTTCTAGGGA | 31510 | 1150 | TGTTCTAGGG |  |
|  |  |  |  |  |  | 4235.59 | 31640 | 31739 | 1449.86 | 31740 | 31839 | 0.342 |  | 29059 | 1053 |  | 29700 | 1017 |  |  |
|  |  |  |  |  |  |  |  |  |  |  |  |  |  | 31451 | 1014 |  | 29348 | 913 |  |  |
| MiSeq | I32 | 157823 | Circular | 1482.64 | 2668.76 | 2898.95 | 30252 | 30351 | 1574.96 | 30352 | 30451 | 0.543 |  | 29498 | 654 | ATATAATAGT | 36918 | 593 | GGTCTACAAT |  |
|  |  |  |  |  |  |  |  |  |  |  |  |  |  | 29114 | 520 |  | 157669 | 465 |  |  |
|  |  |  |  |  |  |  |  |  |  |  |  |  |  | 10821 | 472 |  | 29755 | 458 |  |  |
| MiSeq | I34 | 157851 | Circular | 6301.01 | 11341.82 | 9217.01 | 29000 | 29099 | 19214.61 | 29100 | 29199 | 2.085 |  | 31602 | 3775 | GTTCTAGGGA | 31610 | 2812 | TGTTCTAGGG |  |
|  |  |  |  |  |  | 15297.62 | 30297 | 30396 | 6869.42 | 30397 | 30496 | 0.449 |  | 29159 | 3135 |  | 29448 | 2789 |  |  |
|  |  |  |  |  |  | 22189.93 | 31740 | 31839 | 8324.02 | 31840 | 31939 | 0.375 |  | 31551 | 2794 |  | 29800 | 2612 |  |  |
| MiSeq | I36 | 157701 | Circular | 2687.83 | 4838.09 | 2861.11 | 28995 | 29094 | 5387.68 | 29095 | 29194 | 1.883 |  | 29145 | 697 | GTATAAAGGA | 29434 | 661 | AGTCTTATAC |  |
|  |  |  |  |  |  |  |  |  |  |  |  |  |  | 29529 | 624 |  | 29786 | 648 |  |  |
|  |  |  |  |  |  |  |  |  |  |  |  |  |  | 29426 | 529 |  | 36949 | 642 |  |  |
| MiSeq | I39 | 157715 | Circular | 901.02 | 1621.84 | 1871.55 | 28986 | 29085 | 3676.8 | 29086 | 29185 | 1.965 |  | 29146 | 930 | GTATAAAGGA | 29787 | 985 | ACTTCATGAT |  |
|  |  |  |  |  |  | 3836.39 | 31728 | 31827 | 1185.79 | 31828 | 31927 | 0.309 |  | 31589 | 913 |  | 31597 | 791 |  |  |
|  |  |  |  |  |  |  |  |  |  |  |  |  |  | 29530 | 882 |  | 31827 | 788 |  |  |
| MiSeq | I41 | 157855 | Circular | 1123.39 | 2022.10 |  |  |  |  |  |  |  |  | 29573 | 699 | AGTCTTATAC | 29478 | 654 | ACTATTATAT |  |
|  |  |  |  |  |  |  |  |  |  |  |  |  |  | 29189 | 635 |  | 29830 | 646 |  |  |
|  |  |  |  |  |  |  |  |  |  |  |  |  |  | 29127 | 519 |  | 36993 | 585 |  |  |
| MiSeq | I43 | 157857 | Circular | 2567.46 | 4621.43 | 9753.79 | 31738 | 31837 | 3495.08 | 31838 | 31937 | 0.358 |  | 29540 | 1708 | ATATAATAGT | 29445 | 1394 | AGTCTTATAC |  |
|  |  |  |  |  |  |  |  |  |  |  |  |  |  | 29156 | 1382 |  | 29948 | 1346 |  |  |
|  |  |  |  |  |  |  |  |  |  |  |  |  |  | 31599 | 1158 |  | 29797 | 1324 |  |  |
| MiSeq | I45 | 157713 | Circular | 4586.84 | 8256.32 | 12179.72 | 30239 | 30338 | 4869.41 | 30339 | 30438 | 0.4 |  | 29484 | 2188 | ATATAATAGT | 29741 | 1951 | ACTTCATGAT |  |
|  |  |  |  |  |  | 13229.9 | 31680 | 31779 | 6104.7 | 31780 | 31879 | 0.461 |  | 29100 | 2035 |  | 29389 | 1890 |  |  |
|  |  |  |  |  |  |  |  |  |  |  |  |  |  | 31543 | 1847 |  | 29492 | 1605 |  |  |
| MiSeq | I47 | 157787 | Circular | 2030.77 | 3655.39 | 5602.18 | 30288 | 30387 | 2594.35 | 30388 | 30487 | 0.463 |  | 31592 | 2770 | GTTCTAGGGA | 31600 | 2447 | TGTTCTAGGG |  |
|  |  |  |  |  |  | 6235 | 31731 | 31830 | 1292.95 | 31831 | 31930 | 0.207 |  | 31390 | 1727 |  | 29790 | 1901 |  |  |
|  |  |  |  |  |  |  |  |  |  |  |  |  |  | 31428 | 1658 |  | 29941 | 1597 |  |  |
| MiSeq | I48 | 157912 | Circular | 4042.41 | 7276.34 | 5387.15 | 72762 | 72861 | 9797.35 | 72862 | 72961 | 1.819 |  | 73296 | 1332 | ATATAATAGT | 73201 | 1261 | AGTCTTATAC |  |
|  |  |  |  |  |  | 7137.53 | 74050 | 74149 | 3742.53 | 74150 | 74249 | 0.524 |  | 72912 | 1258 |  | 80716 | 1212 |  |  |
|  |  |  |  |  |  |  |  |  |  |  |  |  |  | 72850 | 1052 |  | 73553 | 1206 |  |  |
| MiSeq | I5 | 157855 | Circular | 2442.58 | 4396.64 | 9282.02 | 74044 | 74143 | 4534.12 | 74144 | 74243 | 0.488 |  | 75350 | 1680 | GTTCTAGGGA | 75358 | 1662 | TGTTCTAGGG |  |
|  |  |  |  |  |  | 6883.42 | 75495 | 75594 | 1151.3 | 75595 | 75694 | 0.167 |  | 73291 | 1413 |  | 73548 | 1580 |  |  |
|  |  |  |  |  |  |  |  |  |  |  |  |  |  | 75148 | 1238 |  | 73699 | 1266 |  |  |
| MiSeq | I8 | 157912 | Circular | 2442.91 | 4397.25 | 8994.41 | 74052 | 74151 | 4096.48 | 74152 | 74251 | 0.455 |  | 75357 | 2357 | GTTCTAGGGA | 75365 | 1945 | TGTTCTAGGG |  |
|  |  |  |  |  |  | 12038.09 | 75499 | 75598 | 2782.52 | 75599 | 75698 | 0.231 |  | 75306 | 1654 |  | 75595 | 1732 |  |  |
|  |  |  |  |  |  |  |  |  |  |  |  |  |  | 72914 | 1614 |  | 73555 | 1634 |  |  |
| MiSeq | I9 | 157856 | Circular | 1165.90 | 2098.62 | 9268.69 | 1323 | 1422 | 4068.06 | 1423 | 1522 | 0.439 |  | 2627 | 2145 | GTTCTAGGGA | 825 | 1759 | ACTTCATGAT |  |
|  |  |  |  |  |  | 8988.98 | 2778 | 2877 | 592.24 | 2878 | 2977 | 0.066 |  | 184 | 1911 |  | 473 | 1668 |  |  |
|  |  |  |  |  |  |  |  |  |  |  |  |  |  | 568 | 1879 |  | 2635 | 1588 |  |  |

Top 3 read edge frequencies were listed. The contig position was bolded if the position was hit in both NCR and read edge frequency within the window. 1.8 FC= 1.8-fold of average coverage, which is the cut-off of NCR method; L= Left window; R= Right window.

**Table S4. Genome terminus prediction of 3 Q8-like isolates using NGS data.**

|  |  |  |  |  |  | NCR |  |  |  |  |  |  |  | Read edge frequency | |  |  |  |  |  |
| --- | --- | --- | --- | --- | --- | --- | --- | --- | --- | --- | --- | --- | --- | --- | --- | --- | --- | --- | --- | --- |
| Sequencer | Isolate | Genome Size | Contig form | Coverage | 1.8 FC | L coverage | L start | L end | R coverage | R start | R end | Coverage ratio |  | 5' edge position | Frequency | flanking sequence | 3' edge position | Frequency | flanking sequence | Size of DTR |
| PGM | Q8 | 158180 | Circular | 115.5256 | 207.9461 | 130.95 | 5026 | 5125 | 236.63 | 5126 | 5225 | 1.807 |  | **156549** | **965** | 5'-AGGTTTTGTG | 156836 | 114 | GCTTCAGAAG-3' | 287 |
|  |  |  |  |  |  | 27.73 | 156449 | 156548 | 1899.41 | **156549** | 156648 | **68.497** |  | 156551 | 475 |  | 156843 | 103 | AAGAATAATA-3' | 294 |
|  |  |  |  |  |  |  |  |  |  |  |  |  |  | 157214 | 331 |  | 156848 | 100 | TAATACATAG-3' | 299 |
|  |  |  |  |  |  |  |  |  |  |  |  |  |  |  |  |  | 156835 | 90 |  | 286 |
|  |  |  |  |  |  |  |  |  |  |  |  |  |  |  |  |  | 156838 | 71 |  | 289 |
|  |  |  |  |  |  |  |  |  |  |  |  |  |  |  |  |  | 158138 | 69 |  | 1589 |
|  |  |  |  |  |  |  |  |  |  |  |  |  |  |  |  |  | 156846 | 62 |  | 297 |
|  |  |  |  |  |  |  |  |  |  |  |  |  |  |  |  |  | 156842 | 60 |  | 293 |
|  |  |  |  |  |  |  |  |  |  |  |  |  |  |  |  |  | **5025** | **56** | TATTTTTCGA-3' | 6656 |
| PGM | Q10 | 158174 | Circular | 377.1076 | 678.7937 | 216.19 | 86302 | 86401 | 1778.86 | **86402** | 86501 | **8.228** |  | **86402** | **730** | 5-'AGGTTTTGTG | 86748 | 194 | GTTATTTGAG-3' | 346 |
|  |  |  |  |  |  |  |  |  |  |  |  |  |  | 86404 | 400 |  | 86737 | 116 | TTCTTTTTTG-3' | 335 |
|  |  |  |  |  |  |  |  |  |  |  |  |  |  | 87067 | 370 |  | **93056** | **99** | TTATTTTTCG-3' | 6654 |
| PGM | Q2 | 158178 | Circular | 360.5248 | 648.9447 | 207.55 | 91198 | 91297 | 1838.6 | **91298** | 91397 | **8.859** |  | **91298** | **597** | 5-'AGGTTTTGTG | 91633 | 146 | TTCTTTTTTG-3' | 335 |
|  |  |  |  |  |  | 746.64 | 93881 | 93980 | 1349.59 | 93981 | 94080 | 1.808 |  | 91300 | 558 | 5'-GTTTTGTGTT | 91644 | 141 | GTTATTTGAG-3' | 346 |
|  |  |  |  |  |  | 497.43 | 97856 | **97955** | 272.14 | 97956 | 98055 | **0.547** |  | 91963 | 286 | 5'-AAGATATTGA | **97953** | **93** | TTATTTTTCG-3' | 6655 |

Top 3 read edge frequencies were listed for Q10 and Q2, whilst top 10 read edge frequencies were listed for Q8. The contig position was bolded if the position was hit in both NCR and read edge frequency within the window. 1.8 FC= 1.8-fold of average coverage, which is the cut-off of NCR method; L= Left window; R= Right window.

**Table S5. Genome terminus prediction of 4 Q11-like isolates using NGS data.**

|  |  |  |  |  |  | NCR |  |  |  |  |  |  |  | Read edge frequency | |  |  |  |  |  |
| --- | --- | --- | --- | --- | --- | --- | --- | --- | --- | --- | --- | --- | --- | --- | --- | --- | --- | --- | --- | --- |
| Sequencer | Isolate | Genome Size | Contig form | Coverage | 1.8 FC | L coverage | L start | L end | R coverage | R start | R end | Coverage ratio |  | 5' edge position | Frequency | flanking sequence | 3' edge position | Frequency | flanking sequence | Size of DTR |
| MiSeq | I17 | 22845 | Linear | 14997.09 | 26994.77 | No hit |  |  |  |  |  |  |  | 1 | 8761 | 5'-GGTGTACATA | 22721 | 6338 |  | N/A |
|  |  |  |  |  |  |  |  |  |  |  |  |  |  | 11931 | 3337 |  | 13814 | 4909 |  |  |
|  |  |  |  |  |  |  |  |  |  |  |  |  |  | 13806 | 3281 |  | 2487 | 3763 |  |  |
| MiSeq | I3 | 21717 | Linear | 5746.133 | 10343.04 | No hit |  |  |  |  |  |  |  | 1 | 7787 | 5-'GCCAACGGAT | 21656 | 2971 | TTTGCATTAC-3' | N/A |
|  |  |  |  |  |  |  |  |  |  |  |  |  |  | 11760 | 1350 |  | 441 | 2175 |  |  |
|  |  |  |  |  |  |  |  |  |  |  |  |  |  | 128 | 1332 |  | 11768 | 1850 |  |  |
| MiSeq | I46 | 24896 | Linear | 15957.76 | 28723.96 | No hit |  |  |  |  |  |  |  | 21829 | 9126 |  | 24896 | 15570 | TAAAAAAGAT-3' | N/A |
|  |  |  |  |  |  |  |  |  |  |  |  |  |  | 1 | 7978 |  | 22142 | 8871 |  |  |
|  |  |  |  |  |  |  |  |  |  |  |  |  |  | 21736 | 7579 |  | 22755 | 6861 |  |  |
| MiSeq | Q11 | 26005 | Linear | 8476.481 | 15257.67 | No hit |  |  |  |  |  |  |  | 15118 | 6717 | 5'-TCCTTACACTG | 15126 | 6090 |  | N/A |
|  |  |  |  |  |  |  |  |  |  |  |  |  |  | 19654 | 4268 |  | 3892 | 3549 |  |  |
|  |  |  |  |  |  |  |  |  |  |  |  |  |  | 20270 | 4032 |  | 20278 | 3460 |  |  |

Top 3 read edge frequencies were listed. The contig position was bolded if the position was hit in both NCR and read edge frequency within the window. 1.8 FC= 1.8-fold of average coverage, which is the cut-off of NCR method; L= Left window; R= Right window.

**Table S6. Genome terminus prediction of SPB8a isolate using NGS data.**

|  |  |  |  |  |  | NCR |  |  |  |  |  |  |  | Read edge frequency | |  |  |  |  |  |
| --- | --- | --- | --- | --- | --- | --- | --- | --- | --- | --- | --- | --- | --- | --- | --- | --- | --- | --- | --- | --- |
| Sequencer | Isolate | Genome Size | Contig form | Coverage | 1.8 FC | L coverage | L start | L end | R coverage | R start | R end | Coverage ratio |  | 5' edge position | Frequency | flanking sequence | 3' edge position | Frequency | flanking sequence | Size of DTR |
| MiSeq | SBP8a | 158822 | Circular | 2522.96 | 4541.32 | 2676 | 29533 | 29632 | 4989.64 | 29633 | 29732 | 1.865 |  | 30078 | 2434 | 5'-ATATAATAGT | 29983 | 2455 | AGTCTTATAC-3' | N/A |
|  |  |  |  |  |  | 6572.65 | 32272 | 32371 | 3413.58 | 32372 | 32471 | 0.519 |  | 59407 | 2021 |  | 30335 | 1958 |  |  |
|  |  |  |  |  |  |  |  |  |  |  |  |  |  | 29975 | 1937 |  | 30086 | 1934 |  |  |
|  |  |  |  |  |  |  |  |  |  |  |  |  |  |  |  |  |  |  |  |  |
| Roche 454 | SBP8a | 158794 | Circular | 87.27 | 157.08 | 239.567 | 111764 | 111793 | 448.9 | **111794** | 111823 | **1.874** |  | **111794** | **117** | 5'-TCAGGTAGAA | 114618 | 371 | AAAAACCTGA-3' | 2822 |
|  |  |  |  |  |  | 779 | 114587 | **114616** | 124.033 | 114617 | 114646 | **0.159** |  | 111796 | 61 | 5'-AGGTAGAACG | **114616** | **238** | AGAAAAACCT-3' |  |
|  |  |  |  |  |  |  |  |  |  |  |  |  |  | 114119 | 36 |  | 114612 | 39 |  |  |

Top 3 read edge frequencies were listed. The contig position was bolded if the position was hit in both NCR and read edge frequency within the window. 1.8 FC= 1.8-fold of average coverage, which is the cut-off of NCR method; L= Left window; R= Right window.

**Table S7. Genome terminus prediction of 9 published isolates using NGS data.**

|  |  |  |  |  |  | NCR |  |  |  |  |  |  |  | Read edge frequency | |  |  |  |  |  |
| --- | --- | --- | --- | --- | --- | --- | --- | --- | --- | --- | --- | --- | --- | --- | --- | --- | --- | --- | --- | --- |
| Sequencer | Isolate | Genome Size | Contig form | Coverage | 1.8 FC | L coverage | L start | L end | R coverage | R start | R end | Coverage ratio |  | 5' edge position | Frequency | flanking sequence | 3' edge position | Frequency | flanking sequence | Size of DTR |
| MiSeq | Adelynn | 162356 | Circular | 215.4328 | 387.779 | 148.81 | 17932 | 18031 | 497.85 | **18032** | 18131 | **3.346** |  | **18032** | **261** | 5'-GGGTTTTTAT | **20724** | **171** | CCGCCTACCC-3' | 2693 |
|  |  |  |  |  |  | 508.3 | 20625 | **20724** | 253.41 | 20725 | 20824 | **0.499** |  | 20574 | 111 |  | 18182 | 148 |  |  |
|  |  |  |  |  |  |  |  |  |  |  |  |  |  | 19366 | 42 |  | 19489 | 43 |  |  |
| Roche454 | Nigalana | 160174 | Circular | 98.05883 | 176.5059 | 118.84 | 6358 | 6457 | 272.83 | **6458** | 6557 | **2.296** |  | **6458** | **121** | 5'-AGGTTTTTCT | **9324** | **95** | CGTTCTACCT-3' | 2867 |
|  |  |  |  |  |  | 212.07 | 9225 | **9324** | 114.93 | 9325 | 9424 | **0.542** |  | 8834 | 63 |  | 6966 | 34 |  |  |
|  |  |  |  |  |  |  |  |  |  |  |  |  |  | 97125 | 14 |  | 31230 | 18 |  |  |
| PGM | Troll | 163019 | Circular | 29.16864 | 52.50355 | no hit |  |  |  |  |  |  |  | 62795 | 7 |  | 43962 | 9 |  | N/A |
|  |  |  |  |  |  |  |  |  |  |  |  |  |  | 32333 | 6 |  | 88320 | 7 |  |  |
|  |  |  |  |  |  |  |  |  |  |  |  |  |  | 146300 | 6 |  | 87302 | 7 |  |  |
| MiSeq | Breeniome | 154434 | Circular | 60.10079 | 108.1814 | no hit |  |  |  |  |  |  |  | 26061 | 17 |  | 48207 | 19 |  | N/A |
|  |  |  |  |  |  |  |  |  |  |  |  |  |  | 1949 | 17 |  | 94014 | 17 |  |  |
|  |  |  |  |  |  |  |  |  |  |  |  |  |  | 31738 | 15 |  | 22652 | 14 |  |  |
| Roche454 | Teardrop | 155389 | Circular | 27.04679 | 48.68423 | 63.26 | 11010 | **11109** | 31.44 | 11110 | 11209 | **0.497** |  | 10666 | 26 |  | **11109** | **28** | CCGCTCCGTT-3' |  |
|  |  |  |  |  |  | 52.73 | 87219 | 87318 | 26.45 | 87319 | 87418 | 0.502 |  | 86912 | 19 |  | 87318 | 21 |  |  |
|  |  |  |  |  |  |  |  |  |  |  |  |  |  | 77060 | 17 |  | 149602 | 17 |  |  |
| PGM | Zeenon | 155292 | Circular | 179.0281 | 322.2506 | no hit |  |  |  |  |  |  |  | 139104 | 23 |  | 10008 | 22 |  | N/A |
|  |  |  |  |  |  |  |  |  |  |  |  |  |  | 153938 | 22 |  | 14389 | 21 |  |  |
|  |  |  |  |  |  |  |  |  |  |  |  |  |  | 26084 | 21 |  | 139787 | 21 |  |  |
| MiSeq | Equemioh13 | 53042 | Circular | 394.3426 | 709.8167 | 86.24 | 40780 | 40879 | 414.22 | **40880** | 40979 | **4.803** |  | **40880** | **276** | 5'-TGCGGCCGCC | 41030 | 191 |  |  |
|  |  |  |  |  |  |  |  |  |  |  |  |  |  | 45395 | 46 |  | 40869 | 56 |  |  |
|  |  |  |  |  |  |  |  |  |  |  |  |  |  | 1073 | 45 |  | 41851 | 43 |  |  |
|  |  |  |  |  |  |  |  |  |  |  |  |  |  |  |  |  |  |  |  |  |
| Roche454 | Zetzy | 48463 | Linear | 80.92188 | 145.6594 | 47.61 | 34429 | 34528 | 158.09 | **34529** | 34628 | **3.321** |  | **34529** | **116** | 5'-CCTGTGCGCC | 34586 | 70 |  | N/A |
|  |  |  |  |  |  |  |  |  |  |  |  |  |  | 42513 | 29 |  | 48463 | 37 |  |  |
|  |  |  |  |  |  |  |  |  |  |  |  |  |  | 245 | 28 |  | 28907 | 25 |  |  |
| PGM | Lilith | 50827 | Circular | 668.8557 | 1203.94 | 412.35 | 5083 | 5182 | 796.31 | 5183 | 5282 | 1.931 |  | 5180 | 89 |  | 3846 | 113 |  | N/A |
|  |  |  |  |  |  |  |  |  |  |  |  |  |  | 32979 | 82 |  | 778 | 82 |  |  |
|  |  |  |  |  |  |  |  |  |  |  |  |  |  | 13025 | 81 |  | 37658 | 65 |  |  |

Top 3 read edge frequencies were listed. The contig position was bolded if the position was hit in both NCR and read edge frequency within the window. 1.8 FC= 1.8-fold of average coverage, which is the cut-off of NCR method; L= Left window; R= Right window.

Table S8. A comparison of genome end characterization between published data on phageDB and predicted result by Terminus package.

| Phage name | Character of genome ends | Curated terminus sequence in phageDB | Length of terminus | Contig form | Predicted terminus sequence from Terminus package (sequence, position on fasta file*) | Predicted length of terminus |
| --- | --- | --- | --- | --- | --- | --- |
| Adelynn | Direct Terminal Repeat | N/A | 2693 bp, LTR | Circular | 5'-GGGTTTTTAT, 18032  CCGCCTACCC-3', 20724 | 2693 bp, LTR |
| Nigalana | Direct Terminal Repeat | N/A | 2867 bp, LTR | Circular | 5'-AGGTTTTTCT, 6458  CGTTCTACCT-3', 9324 | 2867 bp, LTR |
| Troll | Unknown | N/A | N/A | Circular | No Hit | - |
| Breeniome | Circularly Permuted | - | - | Circular | No Hit | - |
| Teardrop | Circularly Permuted | - | - | Circular | CCGCTCCGTT-3', 11109 | - |
| Zeenon | Circularly Permuted | - | - | Circular | No Hit | - |
| Equemioh13 | 3' Sticky Overhang | 5’-CGGTCGGTTA, 40870 | 10 bp, overhang | Circular | 5'-TGCGGCCGCC, 40880^+^ | - |
| Zetzy | 3' Sticky Overhang | 5’-CGGGTGGTAA, 48464 | 10 bp, overhang | Linear | 5'-CCTGTGCGCC, 34529 | - |
| Lilith | 3' Sticky Overhang | 5’-CGGGTGGTAA, 43164 | 10 bp, overhang | Circular | No Hit | - |

* The phage genome sequences in fasta format were downloaded from either <http://phagesdb.org/> or <http://bacillus.phagesdb.org/> as of 1/30/2017.

+ The coverage of overhang and flanking region was plotted in Figure S9.

**Table S9. Primer sequences in primer walking for validating physical ends of phage genomes.**

| Primer name | Sequence (5’ – 3’) | Note |
| --- | --- | --- |
| I48-like-IL | 5'- CGACGTTTATTGACAAGGGTATG -3' | Locate inside of DTR region |
| I48-like-IR | 5'- TGCGTTCTAGGGAGCTACTAA -3' | Locate inside of DTR region |
| I48-like-EL | 5'- GGGCAAATGATACGTTAAAGGAG -3' | Locate outside of DTR region |
| I48-like-ER | 5'- GAGCCTTACTATTAGCCGGAAC -3' | Locate outside of DTR region |
| SBP8a-IL | 5'- TGCGTTCTAGGGAGCTACTAA -3' | Locate inside of DTR region |
| SBP8a-IR | 5'- TATTGTGTCCGGCGTAGTTAAG -3' | Locate inside of DTR region |
| SBP8a-EL | 5'- GCAGACGGAACAAGTGTTCTTA -3' | Locate outside of DTR region |
| SBP8a-ER | 5'- CATGGGTAGAGTTCACAGGTAAT -3' | Locate outside of DTR region |
| Q8-like-IL | 5'- TTAGCAACCTAGCAGCAGAAG -3' | Locate inside of DTR region |
| Q8-like-IR | 5'- GGTGGACAGTACCATTGTTGAG -3' | Locate inside of DTR region |
| Q8-like-EL | 5'- CCTATTACACACCCACCCAAA -3' | Locate outside of DTR region |
| Q8-like-ER | 5'- GGTCTTCGTATGTGGCTTACA -3' | Locate outside of DTR region |
| Q11-like-5'end | 5'- GTGGGTACTGAATATGCCTTGTA -3' | Primer towards 5’ end of Q11 |
| Q11-like-3'end | 5'- CGTATGCTCTTGATGCCATAACT -3' | Primer towards 3’ end of Q11 |
| Q11-like-5'cohesive-14810 | 5'- GGTGAATCACATCGAATGTCAAC -3' | Examine a potential cohesive end of Q11 from 15,118-15,126 |
| Q11-like-3'cohesive-15468 | 5'- GACGTTCCTGCACCCAATAAA -3' | Examine a potential cohesive end of Q11 from 15,118-15,126 |
